# Damage Localization on a Complex Composite Structure Based on NRMSD and Normal Distribution Using Ultrasonic Guided Waves

**DOI:** 10.3390/s26185804

**Published:** 2026-09-13

**Authors:** Houssam El Moutaouakil, Enes Savli, Daniel Lozano, Andreas Schütze

**Affiliations:** 1 Lab for Measurement Technology, Saarland University, 66123 Saarbruecken, Germany; schuetze@lmt.uni-saarland.de; 2 Fraunhofer Institute for Ceramic Technologies and Systems (IKTS), 01109 Dresden, Germany; enes.savli@ikts.fraunhofer.de; 3 Federal Institute for Materials Research and Testing, 12205 Berlin, Germany; daniel-hernando.lozano-duarte@bam.de

**Keywords:** carbon fiber composite plates, damage detection, damage localization, interpretable machine learning, structural health monitoring, ultrasonic guided waves

## Abstract

**Highlights:**

**What are the main findings?**

- Interpretable NRMSD-based damage localization applied to a complex CFRP structure with an integrated omega stringer.
- Transferability of the previously developed localization framework demonstrated with nearly 95% classification accuracy.

**What are the implications of the main findings?**

- Normal-distribution-based localization achieves an average localization error of about 5 mm.
- Continuous spatial coverage achieved while preserving the high localization accuracy of the ellipsoid-based approach.
- Influence of 13 progressively increasing damage sizes on classification and localization performance systematically investigated.

**Abstract:**

Continuous structural health monitoring is essential for ensuring the safe operation of critical engineering systems. Ultrasonic guided waves are widely used for damage detection and localization due to their ability to cover large areas with high sensitivity to structural changes. This work evaluates the robustness of a previously proposed guided-wave localization approach by applying it to the complex geometry of carbon fiber composite plates with integrated omega stringers. The measurement data used in this study were provided within the framework of the Open Guided Waves project. We employ an interpretable machine-learning framework based on the normalized root mean square deviation to extract damage-sensitive features. Damage localization is further improved by modeling the spatial damage probability using a normal distribution, which enhances spatial coverage of the structure. The influence of 13 damages with progressively increasing size on classification and localization performance is systematically analyzed. The proposed method achieves an average classification accuracy of 95% and a mean localization error of 5 mm, demonstrating its suitability for damage characterization in complex composite structures.

## 1. Introduction

Structural health monitoring (SHM) continuously assesses the condition of critical components and supports timely maintenance before failure occurs [1]. Among those components, carbon fiber composites can develop several types of damage, including deformation, delamination, cracking, and fiber breakage [2]. These damage mechanisms are commonly monitored using nondestructive testing (NDT) methods [3]. Ultrasonic guided waves (UGWs) are widely used because they can inspect large structural areas while propagating over long distances in thin-walled structures [4]. UGWs are also sensitive to small structural changes, making them suitable for monitoring safety-critical components [5].

Piezoelectric transducers are typically used to generate and receive UGWs. The same transducer can operate as both a transmitter and a receiver. When one transducer transmits a signal and another receives it, the measurement is referred to as the pitch-catch method [6]. Distributing several transducers across a structure enables continuous monitoring of its condition [7].

Several methods have been proposed for damage detection and localization using ultrasonic guided waves. Time-of-flight methods estimate the damage location from the propagation times of direct or scattered wave components [8,9]. Further approaches combine time-of-flight estimates from multiple measurements to improve anomaly detection and localization [10]. Beamforming methods, such as delay-and-sum imaging, instead use propagation delays to coherently combine sensor signals and reconstruct possible damage locations [11]. These methods generally require reliable identification of damage-related wave components and sufficiently accurate knowledge of the wave propagation characteristics.

Another established group of methods uses damage indicators obtained by comparing baseline and damaged signals. The reconstruction algorithm for probabilistic inspection of damage (RAPID) evaluates changes in signal correlation and assigns the resulting damage indices to elliptical spatial distributions [12]. The contributions of several propagation paths are then combined to reconstruct tomograms that indicate possible damage locations. Several extensions of RAPID have been proposed to improve the spatial weighting and increase the robustness of the localization results [13,14]. Baseline-free damage imaging methods have also been developed to localize damage from guided-wave measurements without requiring a reference measurement from the undamaged structure [15]. Other approaches formulate damage localization as an inverse problem. Sparse-reconstruction methods estimate damage-related spatial distributions from a limited number of measurements, while model-based imaging methods use analytical or numerical representations of wave propagation to reconstruct possible damage locations [16,17].

The different approaches rely on different information about the investigated structure and measured wave responses. Time-of-flight and beamforming methods depend on propagation times or wave-field information, whereas sparse and model-based reconstruction methods require assumptions about the spatial damage representation or the underlying propagation model. Path-based tomographic approaches such as RAPID are particularly suitable when baseline measurements and signals from a distributed sensor network are available. They evaluate changes along individual transmitter-receiver paths and transfer the resulting path information to the spatial domain.

More recently, machine-learning (ML)-based approaches have been proposed [5]. Deep learning methods, such as the approach described in [18,19], have demonstrated promising performance; however, they often require large datasets and may overfit when trained on limited experimental data [20]. In addition, their limited interpretability poses challenges for safety-relevant SHM applications [21]. Among the modified localization approaches, ellipsoid-based reconstruction methods exploit the geometric relationship between the transmitter, receiver, and damage location to improve localization accuracy [22]. The interpretable ML approach based on the normalized root mean square deviation (NRMSD) belongs to this class and evaluates interval-wise differences between baseline and damaged signals [23]. This method was previously developed and validated on a composite overwrapped pressure vessel [24]. By preserving spatial information, the damage location can be reconstructed using ellipsoids whose perimeters intersect at the actual defect position. Although this method achieves high localization accuracy, the ellipsoid-based reconstruction may introduce blind areas that reduce spatial coverage [24].

Although the aforementioned approaches have significantly advanced guided-wave damage localization, they exhibit complementary advantages and limitations. Probabilistic tomographic methods such as RAPID provide continuous spatial coverage but generally produce diffuse localization regions. In contrast, the previously developed ellipsoid-based localization method uses the predicted geometrical relation between individual propagation paths and the damage position and can achieve considerably higher localization accuracy, but may introduce blind areas depending on the sensor network geometry. Path-based localization remains affected by the spatial coverage of the sensor network, intersections between propagation paths, and the representation used to transfer the individual path contributions to the localization map [25]. The different spatial representations therefore provide a suitable basis for investigating the trade-off between localization accuracy and spatial coverage. Consequently, there remains a need for a localization approach that retains the geometrical information provided by the machine learning model while providing continuous spatial coverage.

The NRMSD feature extraction, SVM classification, and ellipsoid-based localization framework were developed in our previous work [24]. The present study builds on this framework by evaluating its transferability to a complex CFRP structure with an integrated omega stringer and by extending the spatial reconstruction approach. Furthermore, the localization strategy is extended by introducing a probabilistic reconstruction based on normal distributions. Whereas the previously proposed ellipsoid-based reconstruction determines the damage location from discrete ellipsoid intersections and may introduce blind areas depending on the sensor geometry, the proposed method reconstructs a continuous probabilistic tomogram. Unlike RAPID approaches, which reconstruct damage probability maps from correlation-based damage indices assigned to elliptical probability distributions [26], the proposed approach incorporates machine-learning-derived localization information into the probabilistic reconstruction. Consequently, it combines the localization accuracy of the ellipsoid-based method with the continuous spatial coverage characteristic of probabilistic tomographic approaches. Finally, the influence of damage size on the localization performance is systematically investigated using an experimental dataset comprising 13 progressively increasing damage sizes [27].

The paper first presents the experimental setup, then describes the developed ML methods. Finally, the obtained results are presented and discussed.

## 2. Design of Experiments

**Figure 1:**
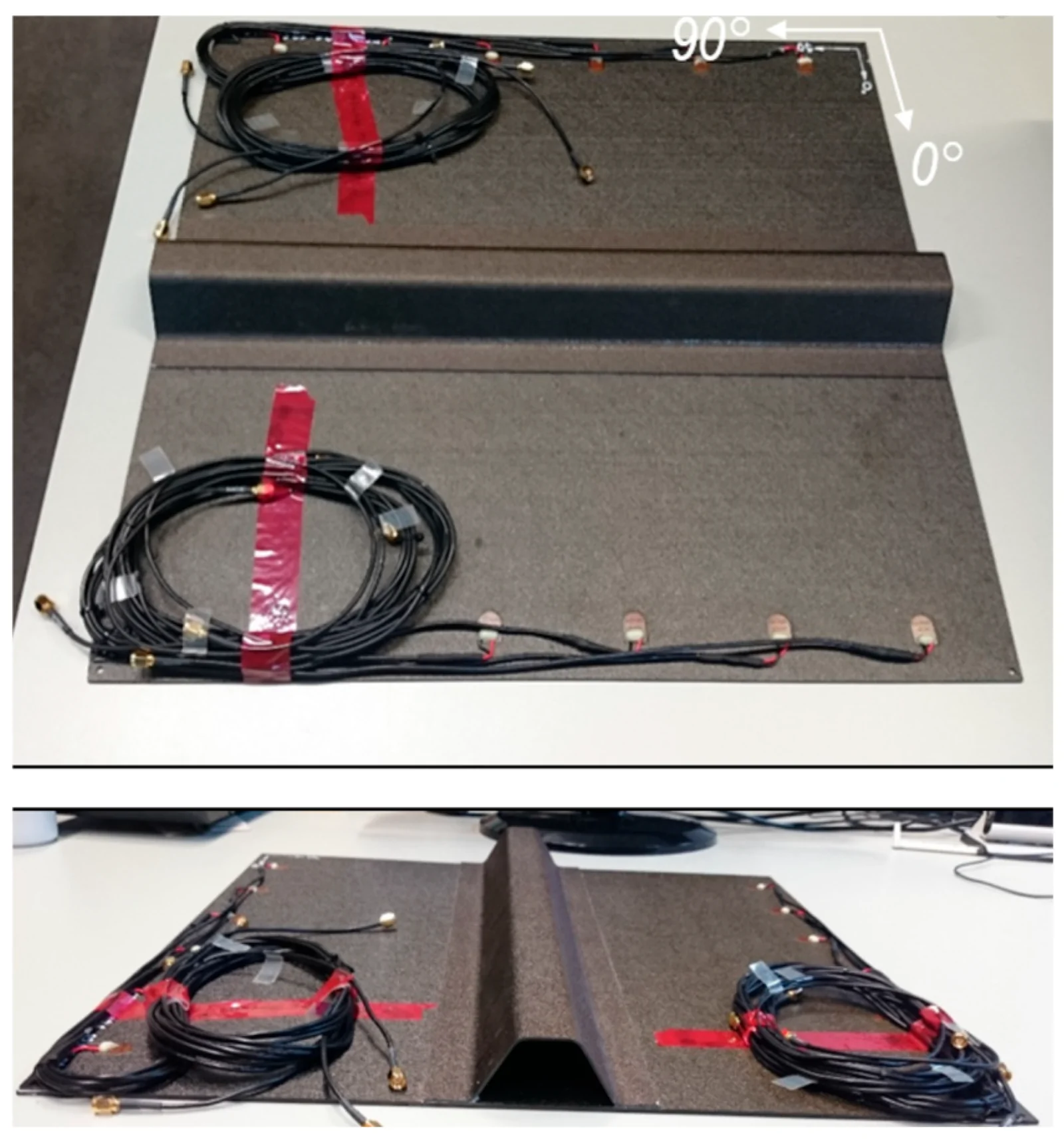
Illustration representing the top and the side view of the CFRP plate, as well as the mounted ultrasonic transducers [27].

The measurements were performed within the scope of the Open Guided Waves project [27]. The experiments conducted to generate the dataset involved a carbon fiber-reinforced polymer (CFRP) plate with an integrated omega stringer structure (Figure 1).

**Figure 2:**
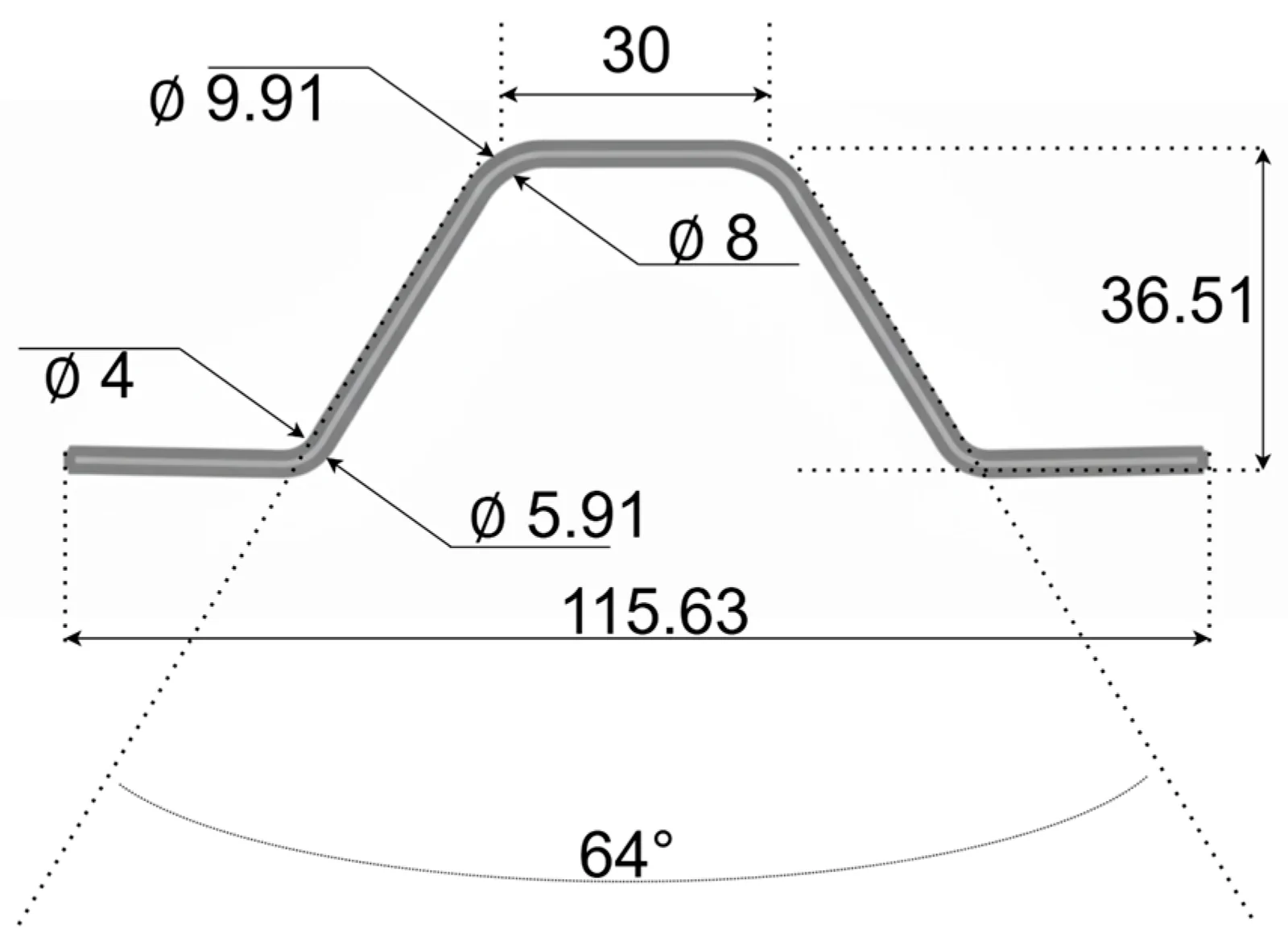
Technical drawing of the cross-section of the omega stringer that is integrated in the CFRP plate [27].

The plate was manufactured from HexPly prepreg material, with a nominal thickness of 2 mm and dimensions of 550 mm × 550 mm. The omega stringer has a nominal thickness of 1.5 mm and is adhesively bonded to the surface of the CFRP plate. Figure 2 illustrates the cross-sectional drawing of the stringer.

**Figure 3:**
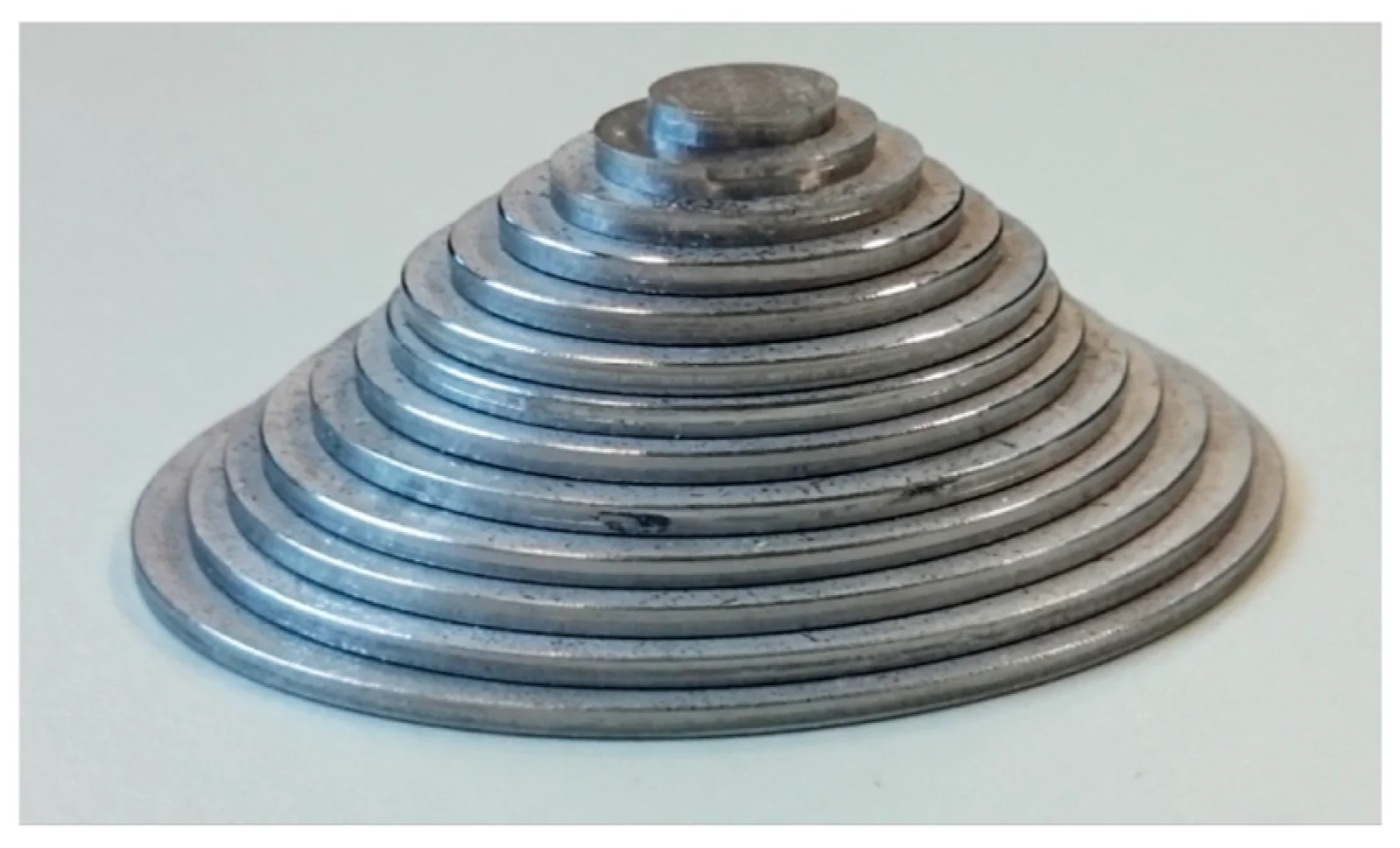
Artificially introduced damages of varying sizes [27].

**Figure 4:**
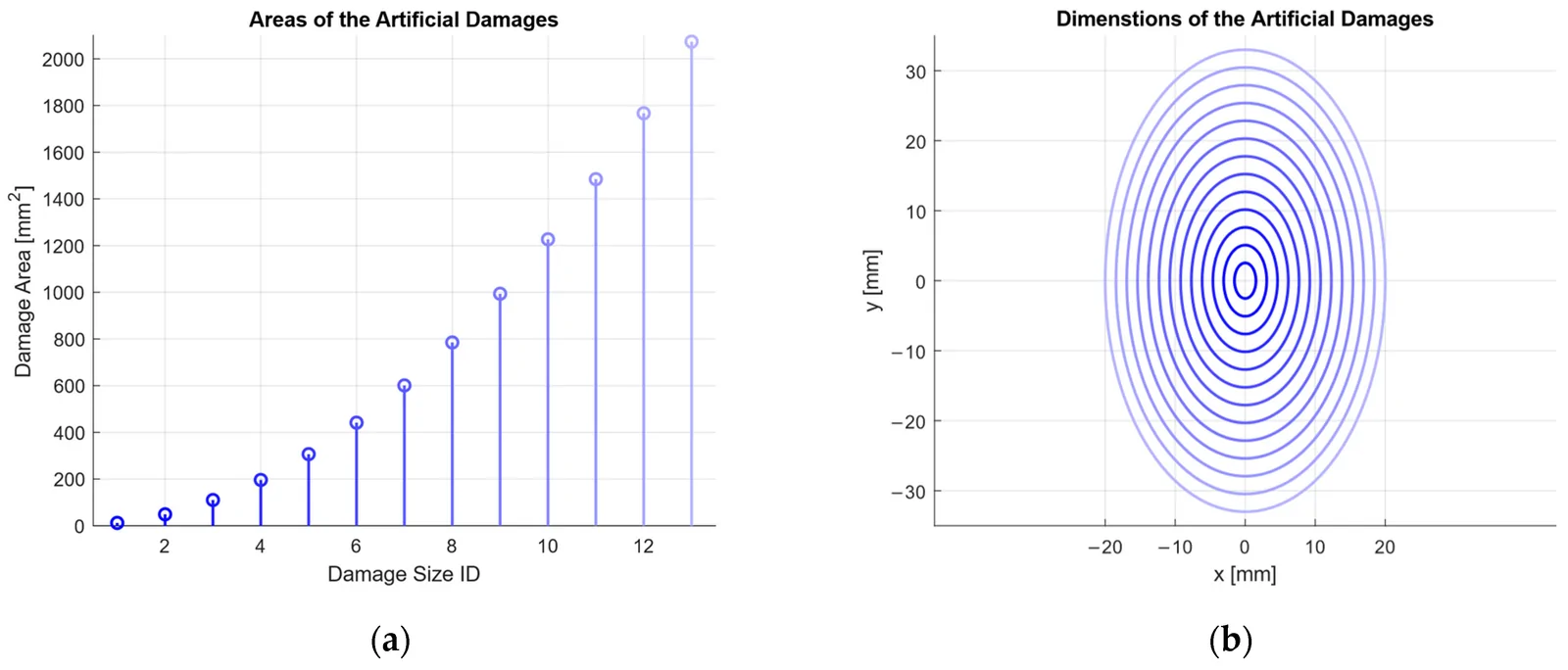
(**a**) Damage areas plotted against the size IDs. (**b**) Corresponding dimensions of the artificial damages [27].

Reversible artificial damages, consisting of 13 elliptically shaped metallic discs (Figure 3), are attached to the CFRP surface using double-sided adhesive tape. The elliptical shape is used to approximate the spatial extent of impact-induced damage. However, these attached masses do not reproduce the internal material changes associated with real delamination or impact damage. Their interaction with guided waves is mainly caused by local mass loading and coupling at the plate surface, which can differ from the scattering caused by stiffness reduction, ply separation, or cracking in a damaged laminate. The artificial damages therefore provide controlled and repeatable test conditions, but the resulting localization performance should not be interpreted as directly equivalent to that for real composite damage. The elliptical shape is intended to represent typical impact-induced damage. Figure 4 illustrates the disc dimensions and their corresponding areas.

**Figure 5:**
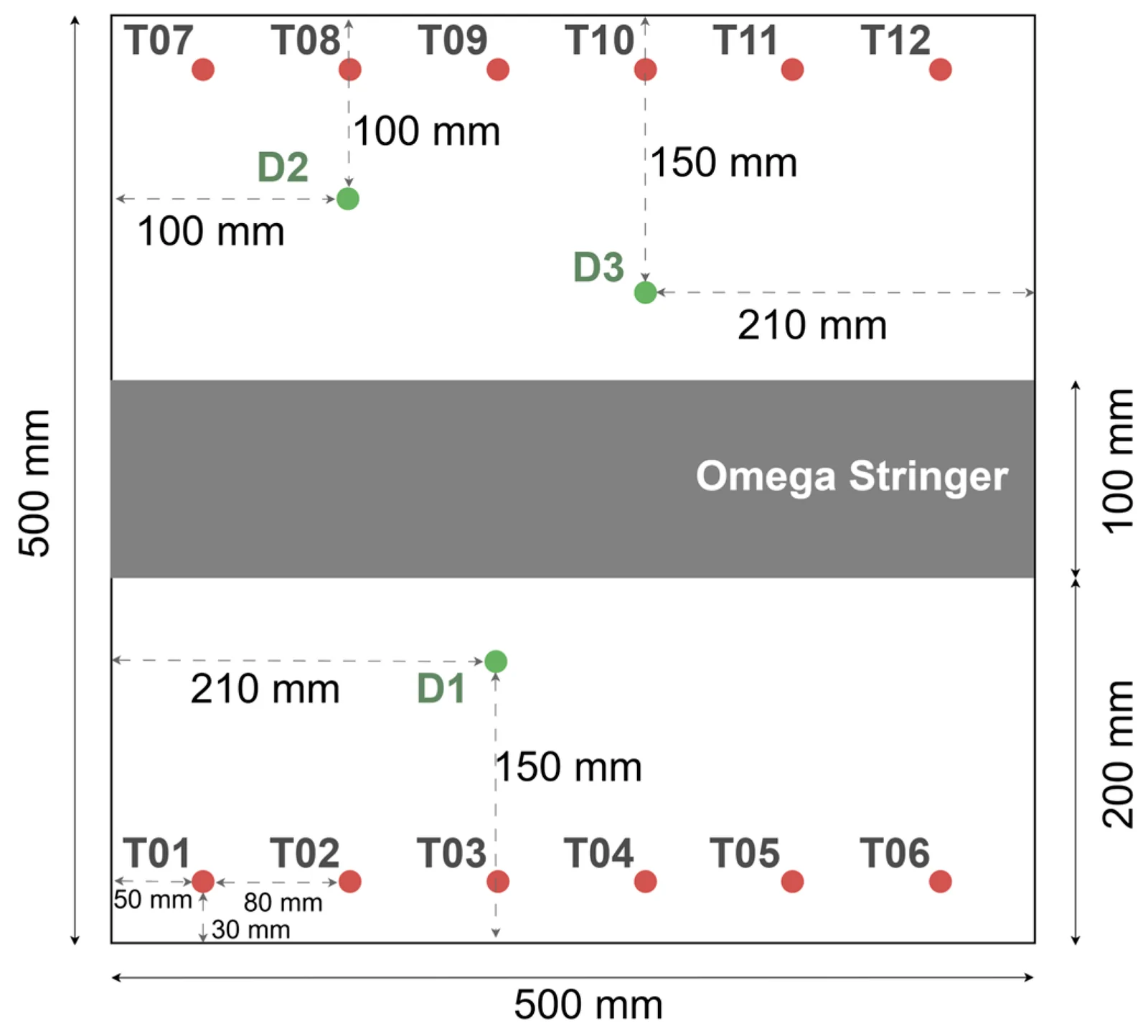
Illustration showing the exact transducer positions and damage location Red markers indicate the transducer positions, while green markers indicate the damage positions [27].

Three damage positions are investigated separately as in Figure 5, with the exact coordinates of each position indicated in the same figure. The experiments are then conducted by placing the CFRP plate in a climate chamber maintained at 23 °C and 50% relative humidity.

The plate is equipped with an array of 12 piezoelectric ultrasonic transducers that act as both transmitters and receivers following the pitch–catch procedure (Figure 5).

**Figure 6:**
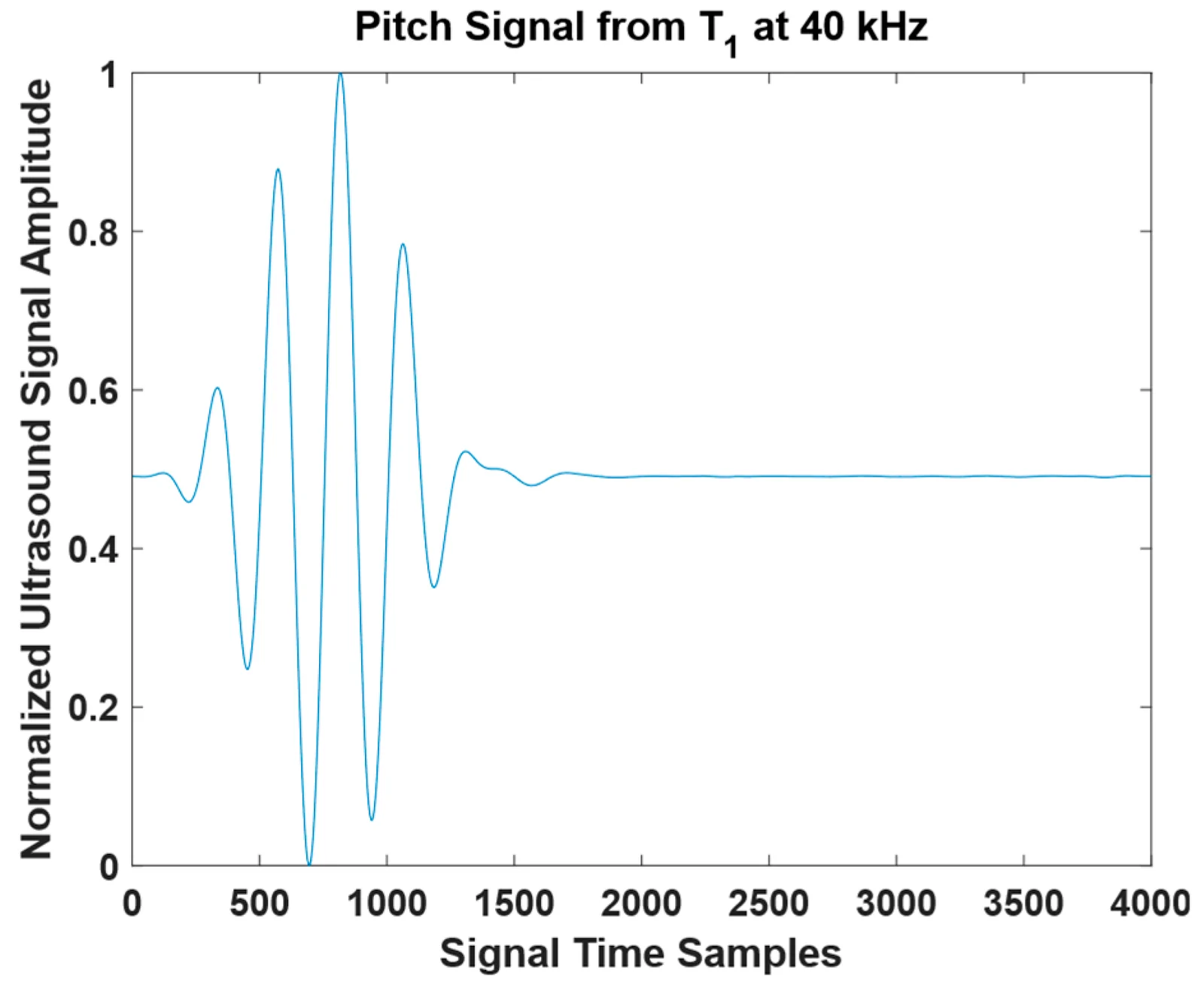
Excitation signal generated by the Transducer T1, consisting of a five-cycle sinusoidal waveform [27].

The UGW signal excitation is performed using piezoceramic transducers, driven by a Handyscope HS5 (TiePie Engineering, Sneek, The Netherlands) and a broadband amplifier PD200 (PiezoDrive Pty Ltd, Newcastle, NSW, Australia), to generate and record ultrasonic waves. The generated excitation signal consists of a sinusoidal signal with five cycles that has been Hann-windowed w(t) to reduce spectral leakage and provide a cleaner mode excitation (Figure 6) [28].

The excitation signal is represented by the following equation:

(1) $$V\left(t\right)=\hat{V}\;w\left(t\right)\;sin(2\pi F_{e}t)$$

The signal amplitude $\hat{V}$ is set to 100 V. The excitation frequency $F_{e}$ is varied from 40 kHz to 260 kHz in steps of 20 kHz. In addition, a broadband chirp signal is applied.

The described experimental setup results in the following number of measurements:

(2) $$223080=F_{e}\times C\times R\times D_{s}\times S_{T}$$

$F_{e}$: 13 excitation frequencies
C: Four plate conditions: undamaged state plus three damage locations (1, 2, 3)
R: Five repetitions per plate condition to generate more data and test reproducibility
$D_{s}$: 13 damage sizes
$S_{T}$: 66 sensor transducer paths (12 × 11/2)

Although measurements were acquired at several excitation frequencies, the following analysis considers only the measurements obtained at 40 kHz (17,160 measurements, of which 12,870 are damage data and 4290 are baseline data). For dispersive guided waves, the wavelength of a specific mode is determined by its phase velocity $c_{p}$, while the group velocity governs the propagation of the wave packet and therefore its arrival time. In anisotropic CFRP laminates, these velocities can additionally depend on the propagation direction. The relation according to Equation (3), is therefore used here as an approximate detectability criterion rather than as a general physical limit [29]. Based on the average sound velocity of 3042 m/s in the transverse direction (90°) reported in [30], this criterion gives an approximate minimum detectable damage size of about 2 cm. At this frequency $F_{e}$, the first antisymmetric mode $A_{0}$ exhibits a low group velocity, which increases the time separation between initial wave arrival, reflections, and mode conversions [31]. Another reason for selecting this frequency is related to planned future work, which aims to compare model accuracy between simulated and experimental data, as simulation data are currently available only at this frequency [32].

(3) $$S_{min}\approx \frac{\lambda }{4}=c/4\;F_{e}$$

## 3. Machine Learning Methods

### 3.1. Description

This study investigates the transferability of a machine-learning method originally developed for damage detection in composite overwrapped pressure vessels [24] to a complex structure consisting of a CFRP plate with an integrated stringer [27]. Furthermore, the influence of damage size on model performance and localization accuracy is systematically analyzed. The proposed ML algorithm follows an interpretable workflow comprising feature extraction, feature selection, and classification (FESC) [33].

In the first step, the UGW signals are smoothed using a second-order Butterworth bandpass filter with a bandwidth of ±15 kHz around the excitation frequency [34].

**Figure 7:**
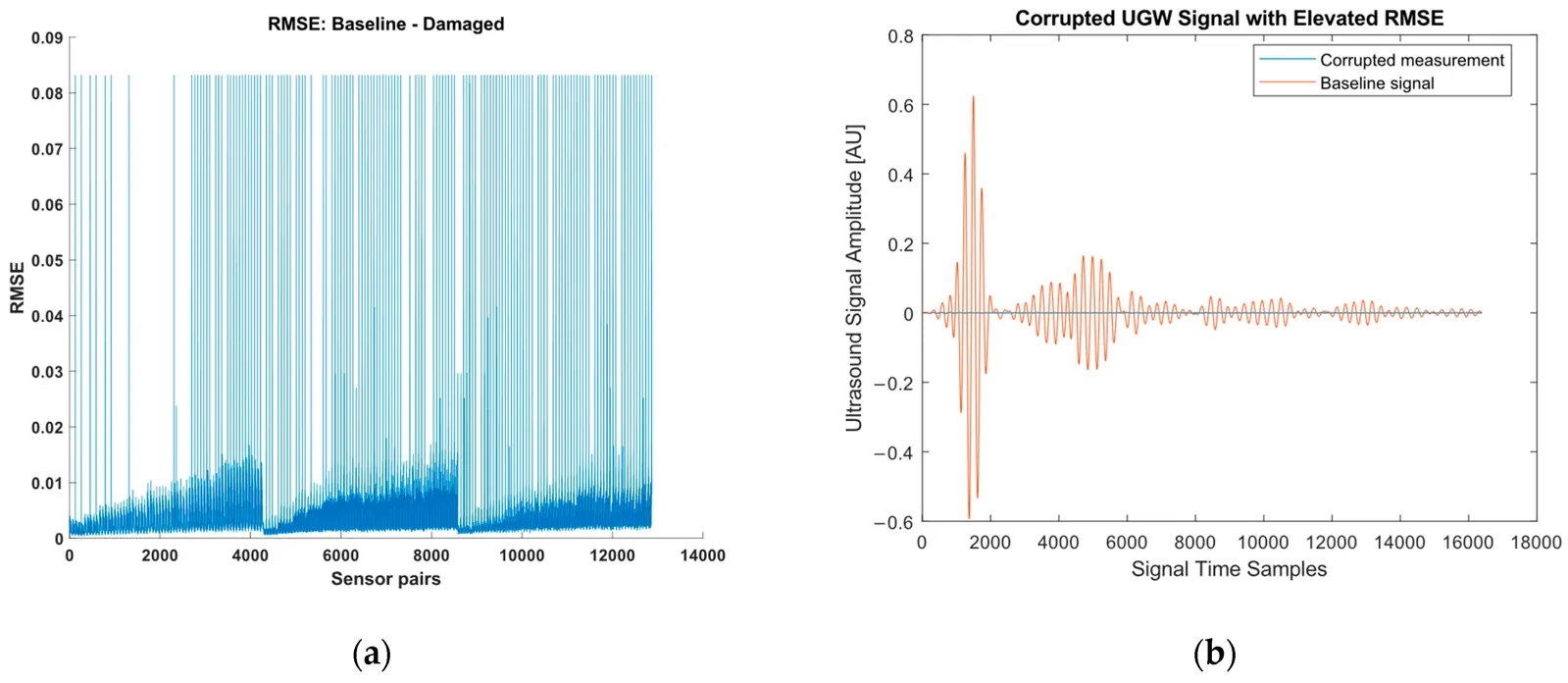
RMSE-based identification of erroneous measurements. (**a**) RMSE values obtained for the investigated measurements, with the threshold set to 0.02. (**b**) Example of a measurement (index 127) with elevated RMSE showing a corrupted UGW waveform compared with the corresponding baseline signal.

**Figure 8:**
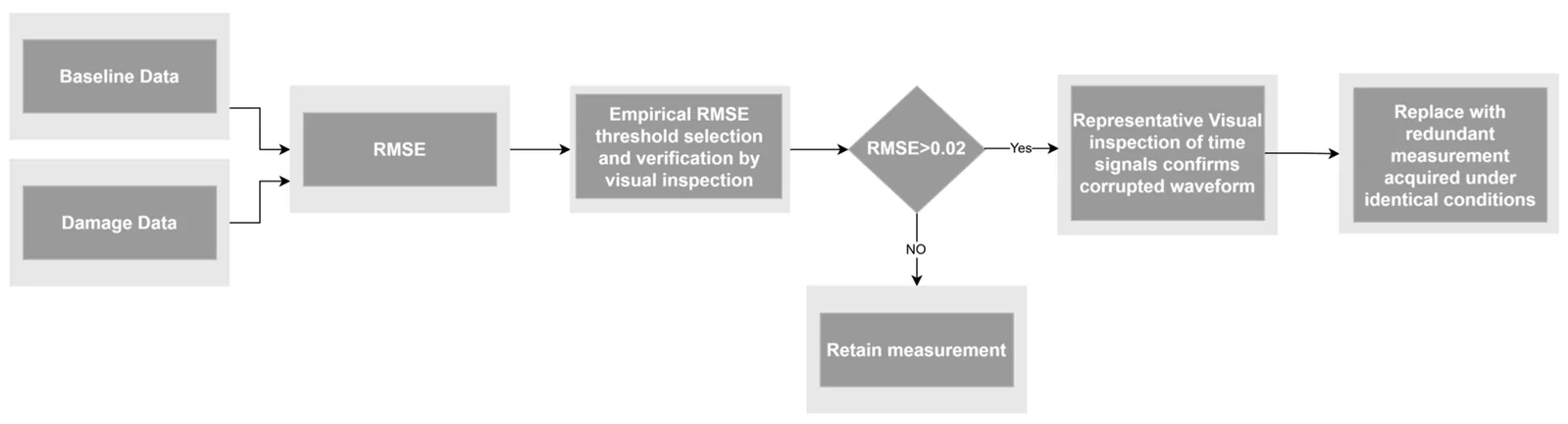
Flowchart of the RMSE-based quality-control procedure for identifying and replacing corrupted measurements.

Quality control and preprocessing of measurements are common practices in structural health monitoring to prevent measurement artefacts from being interpreted as structural changes [35]. Subsequently, signal quality is assessed by comparing damaged and baseline signals using the root mean square error (RMSE) metric (Figure 7a) [36]. Measurements affected by acquisition errors are identified by abnormally high RMSE values. The RMSE distribution showed that the majority of measurements remained clearly below 0.02, whereas corrupted measurements produced distinctly higher values. Therefore, 0.02 was selected as a conservative threshold above the normal RMSE range. Representative measurements exceeding this threshold were inspected in the time domain and showed clear acquisition artefacts (Figure 7b). Measurements above the threshold were then replaced by redundant measurements acquired under identical experimental conditions (Figure 8). These corrupted measurements originated from occasional transient measurement failures rather than structural changes. They were replaced by corresponding redundant measurements acquired under identical experimental conditions. Without this correction, corrupted baseline or damaged signals would produce artificially large signal differences and consequently unrealistically high RMSE values unrelated to structural damage. The redundant measurements were employed solely for this purpose and were not included as independent training or validation samples. Therefore, the original measurement indexing and sensor-path configuration were preserved without introducing additional information into the machine-learning dataset or affecting the statistical independence between the training and evaluation data. Overall, only 158 of the 12,870 acquired measurements (1.23%) exceeded the RMSE threshold and required replacement. Both corrupted baseline and damaged measurements were observed. Since only a small fraction of the complete dataset was affected, the correction served exclusively to remove obvious acquisition artifacts while preserving the statistical characteristics of the experimental dataset.

**Figure 9:**
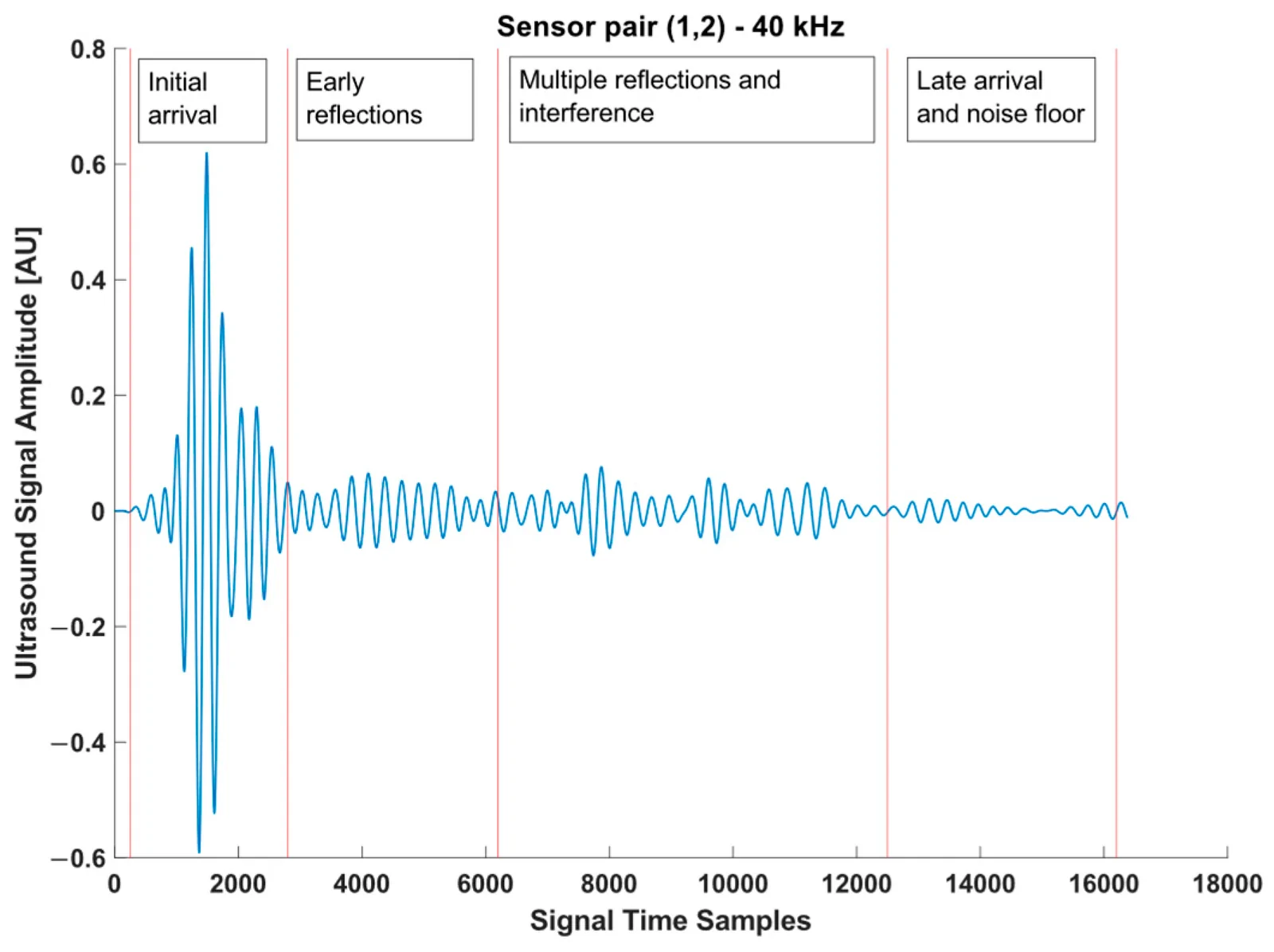
UGW Signal segmented into corresponding arrival regions.

Next, windowing is applied to the signals to refine the region of interest by selecting the time interval corresponding to the initial arrival of the excitation signal (Figure 9) [37].

The feature extraction step is then performed by segmenting both the damaged signal and the corresponding baseline signal into equal time segments. For each pair of corresponding segments, the NRMSD is calculated according to the following equation [38]:

(4) $${NRMSD}_{n}=\frac{\sqrt{\frac{\sum_{t=(n-1)*L+1}^{n*L}{\left(S_{t}-{\hat{S}}_{t}\right)}^{2}}{L}}}{max(S)-min(S)}$$

n: Index of the investigated segment
t: Index of time sample within the investigated segment
L: Length of the investigated segment
S: Baseline signal
$\hat{S}$: Signal affected by damage

**Figure 10:**
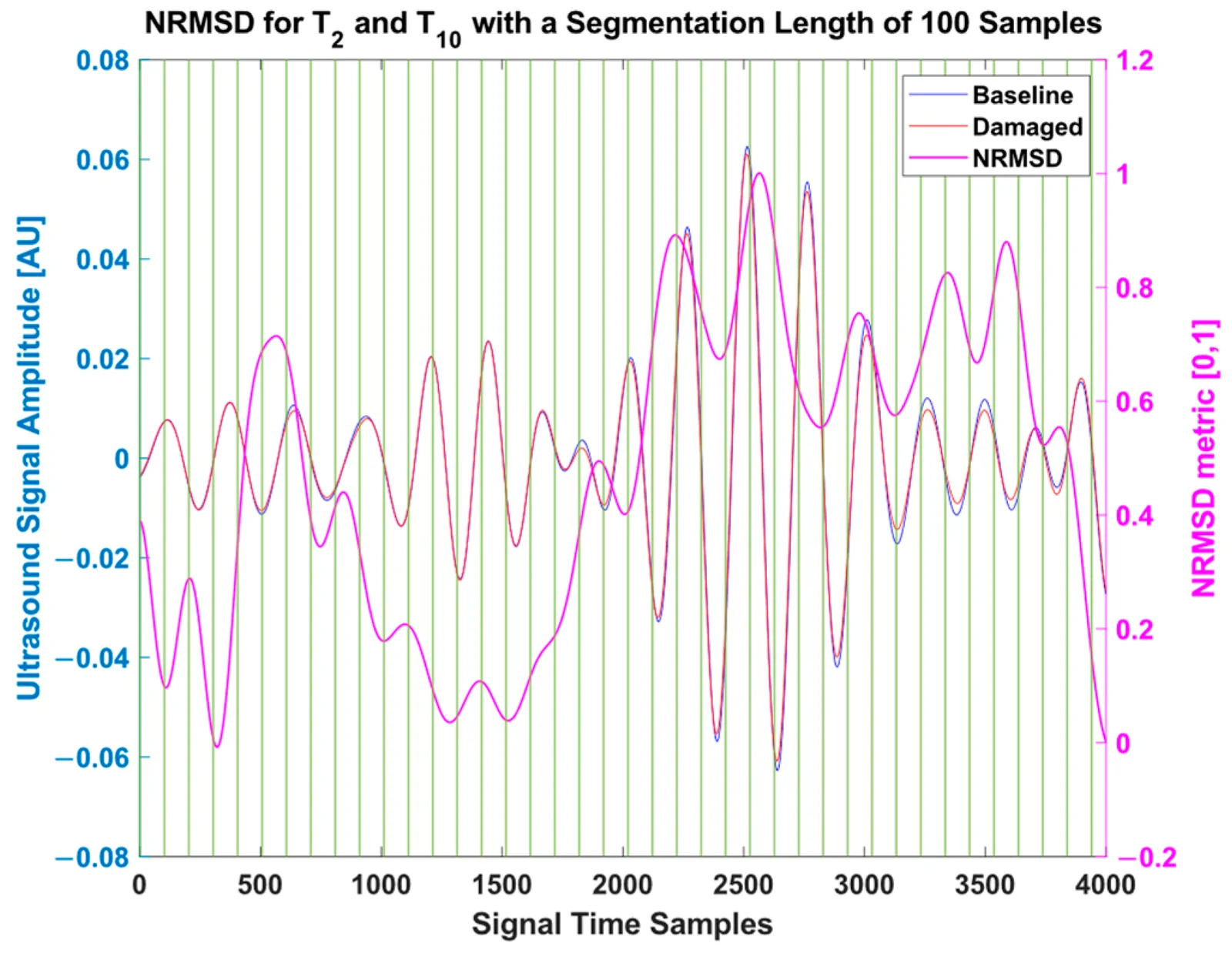
Illustration of the signal segmentation and the corresponding NRMSD features.

Figure 10 illustrates an example of feature extraction based on the NRMSD with a segmentation length of 100 samples. At signal locations where the baseline differs significantly from the damaged signal, the NRMSD values approach 1. In contrast, where the baseline and damaged signals are nearly identical, the NRMSD values approach 0. The optimization of the segmentation length is investigated in the Results section.

**Figure 11:**
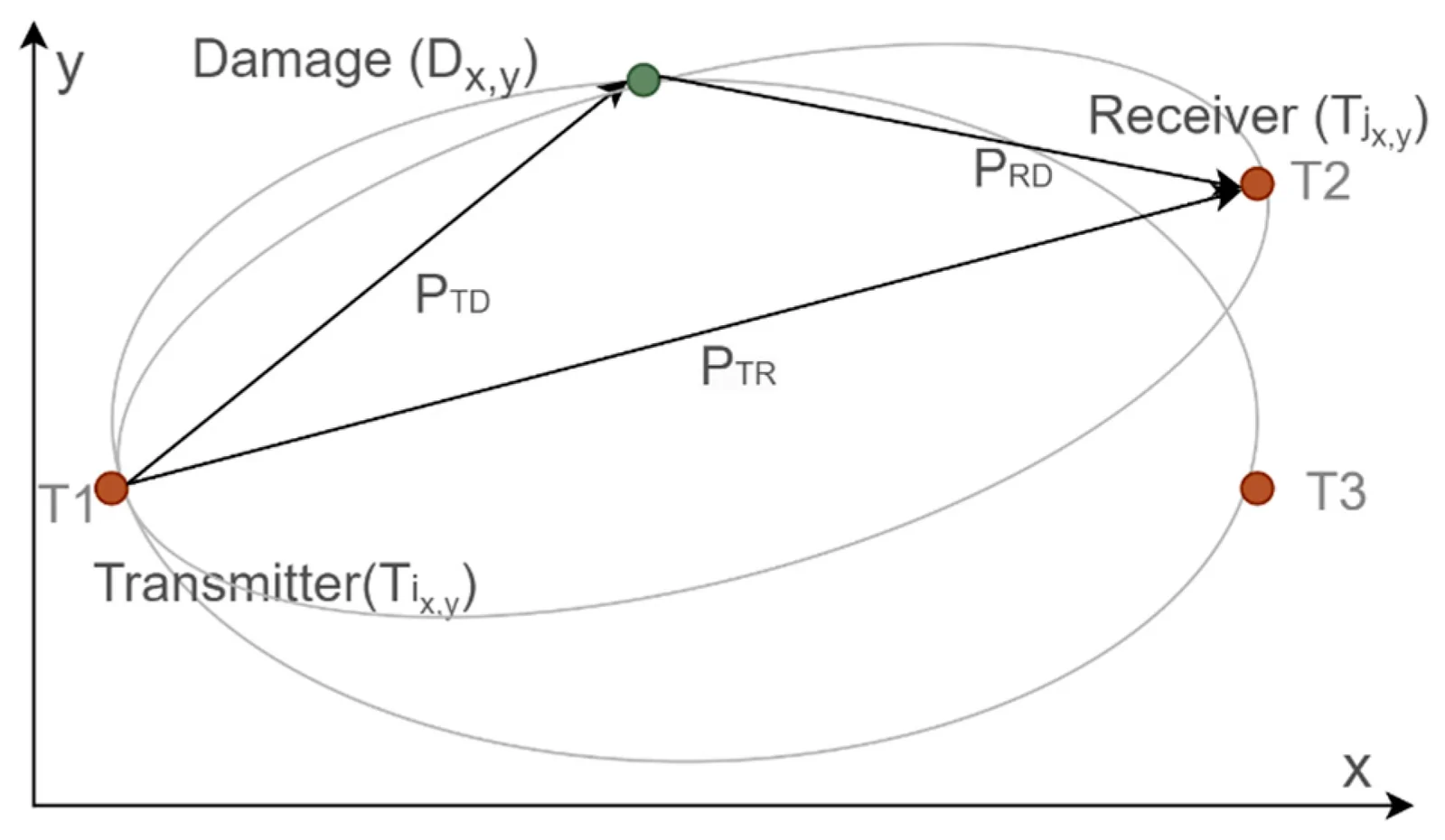
Illustration showing the direct path $P_{TR}$ and indirect path $P_{TD},P_{DR}$ that are used to calculate the diameter ratio.

The model training is performed using quantized target values of the diameter ratio $R_{\emptyset }$, cf. Equation (5), which represents the ratio between the direct path connecting two transducers and the indirect path that originates at the transmitter, passes through the damage, and reaches the receiver (Figure 11) [39]. A ratio close to 1 indicates that the damage lies on or near the direct propagation path, whereas a ratio close to 0 represents damage far from the direct path indicating little to no influence of the damage on the respective sensor pair data. For classification purposes, $R_{\emptyset }$ values below 0.9 are ignored and only the $R_{\emptyset }$ interval from 0.9 to 1 is discretized into 10 equal subintervals to define the model classes [24].

(5) $$R_{\emptyset }=\frac{P_{TR}}{P_{TD}+P_{RD}}=\frac{\sqrt{{\left(x_{T}-x_{R}\right)}^{2}+{\left(y_{T}-y_{R}\right)}^{2}}}{\sqrt{{\left(x_{T}-x_{D}\right)}^{2}+{\left(y_{T}-y_{D}\right)}^{2}}+\sqrt{{\left(x_{R}-x_{D}\right)}^{2}+{\left(y_{R}-y_{D}\right)}^{2}}}$$

$R_{\emptyset }$: Diameter ratio of the ellipsoid for the path from transmitter T to receiver R via damage
$P_{TR}$: Direct path of the ultrasonic wave starting at transducer T and arriving at receiver R
$P_{TD}$: Ultrasonic wave propagation path starting at transducer T and arriving at damage D
$P_{RD}$: Ultrasonic wave propagation path from damage D to receiver R
($x_{T},y_{T}$): Coordinates of the transducer position
($x_{R},y_{R}$): Coordinates of the receiver position
($x_{D},y_{D}$): Coordinates of the damage position

The subsequent step involves normalizing the features, as classification with a support vector machine (SVM) is sensitive to feature scaling [40]. The SVM-based classification task consists of predicting the quantized damage-ratio classes depending on the extracted features for each sensor path.

**Figure 12:**
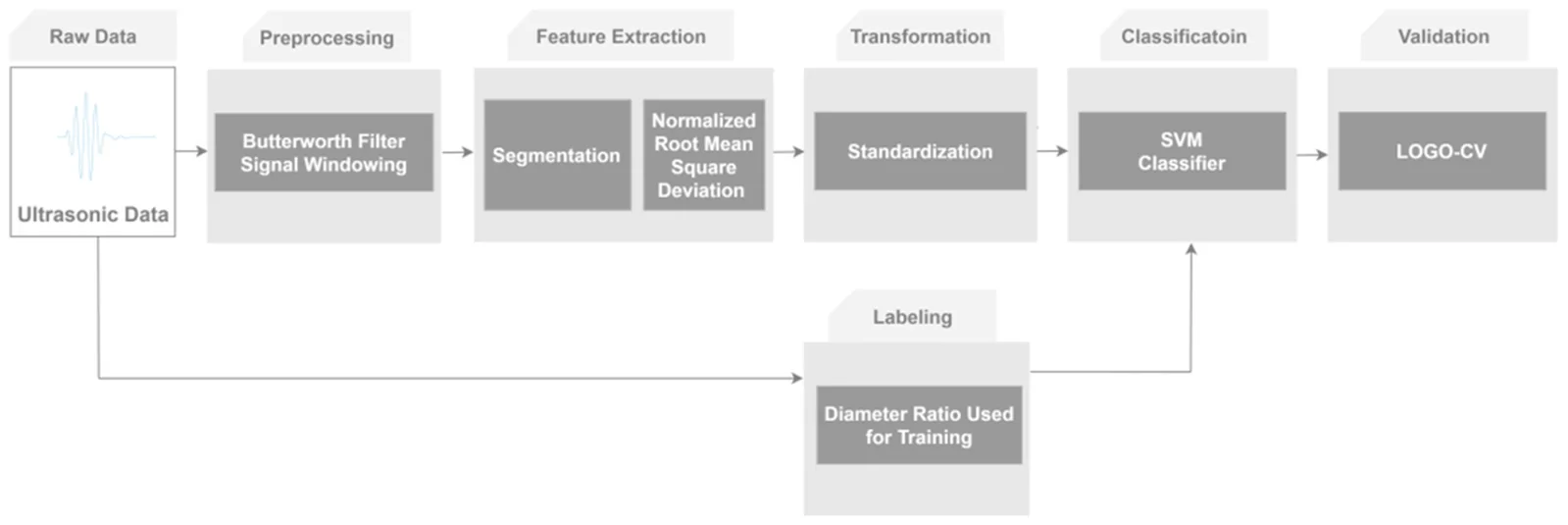
Overview of the processing chain for the proposed machine learning algorithm.

Following that, Leave-One-Group-Out Cross-Validation (LOGO-CV) is used to validate the model. Since the objective is to investigate the influence of damage size on model performance, each validation group corresponds to one complete damage size. Consequently, all measurements associated with the excluded damage size, including all repetitions and sensor-path measurements, are reserved exclusively for testing, while the model is trained only on the remaining damage sizes. This validation strategy prevents data leakage between the training and test sets with respect to the investigated damage size and enables an unbiased evaluation of the model’s ability to generalize to previously unseen damage severities [41]. Figure 12 provides an overview of the complete processing chain described above.

### 3.2. Tomographic Localization

After predicting the damage ratios, different tomographic localization methods are compared to identify the most suitable approach.

**Figure 13:**
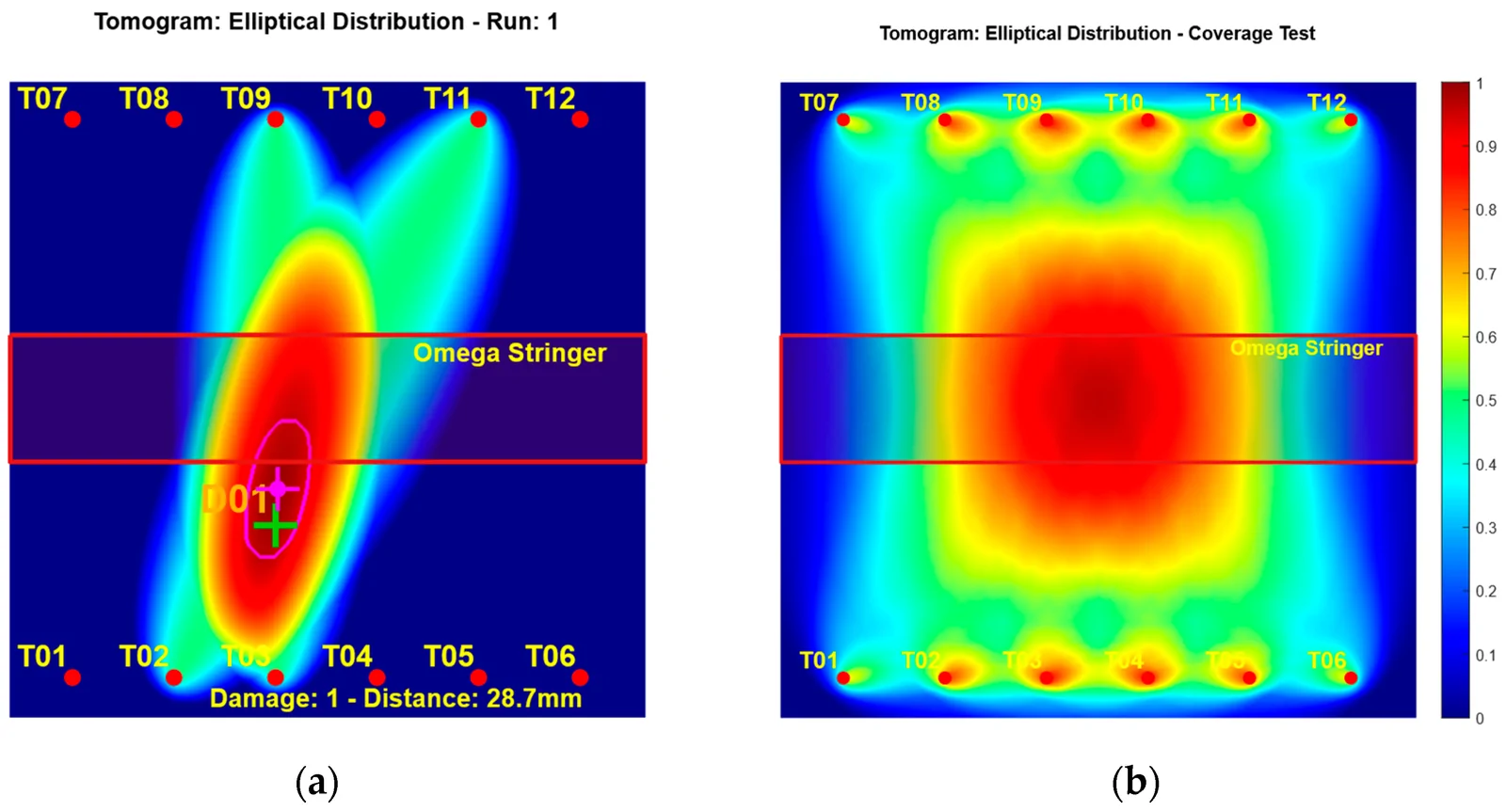
(**a**) Example localization using the elliptical distribution method. (**b**) Corresponding coverage test.

The first method is based on an elliptical distribution, which is commonly used in combination with RAPID. In this approach, ellipses are generated for sensor pairs with diameter ratios greater than the predefined threshold β = 0.9 [42]. The threshold suppresses sensor pairs with low diameter ratios, whose broad ellipses provide only limited localization information while increasing the spatial uncertainty of the tomographic reconstruction. The selected threshold was adopted from the previously proposed ellipsoid-based localization framework [24], where it was shown to provide a suitable compromise between localization accuracy and spatial coverage. Each ellipse is assigned an intensity corresponding to the predicted quantized value, as defined in Equation (5). Figure 13 presents an example of the resulting localization as well as a coverage test illustrating the case in which all sensor paths are influenced by damage.

The elliptical distribution is obtained by thresholding the diameter ratio using the predefined threshold β, as defined in Equation (6). The final tomogram is constructed by superimposing the elliptical distributions calculated for all sensor pairs [42]. For the localization estimate, the connected high-intensity region around the maximum of the reconstructed tomogram is extracted. Pixels with intensity values between 250 and 255 are considered, corresponding to approximately 98% to 100% of the maximum intensity. The coordinates of the centroid are calculated by averaging the corresponding x and y coordinates, following the procedure described in [24]. The coordinates of the centroid are calculated by averaging the corresponding x and y coordinates. The localization error is then calculated as the Euclidean distance between the estimated and actual damage positions. The same procedure is used for all localization methods evaluated in this study.

(6) $$D_{i,j}\left(x,y\right)=\left\{\begin{array}{cc} \frac{\beta -R_{\emptyset }}{\beta -1} & for\;R_{\emptyset }>\beta \\ 0 & else \end{array}\right.$$

D: Reconstructed elliptical shape based on elliptical distribution
$R_{\emptyset }$: Diameter ratio
i,j: Indices of the transmitter-receiver pair i and j
x,y: Cartesian coordinates on the SHM plate
β: Thresholding parameter

**Figure 14:**
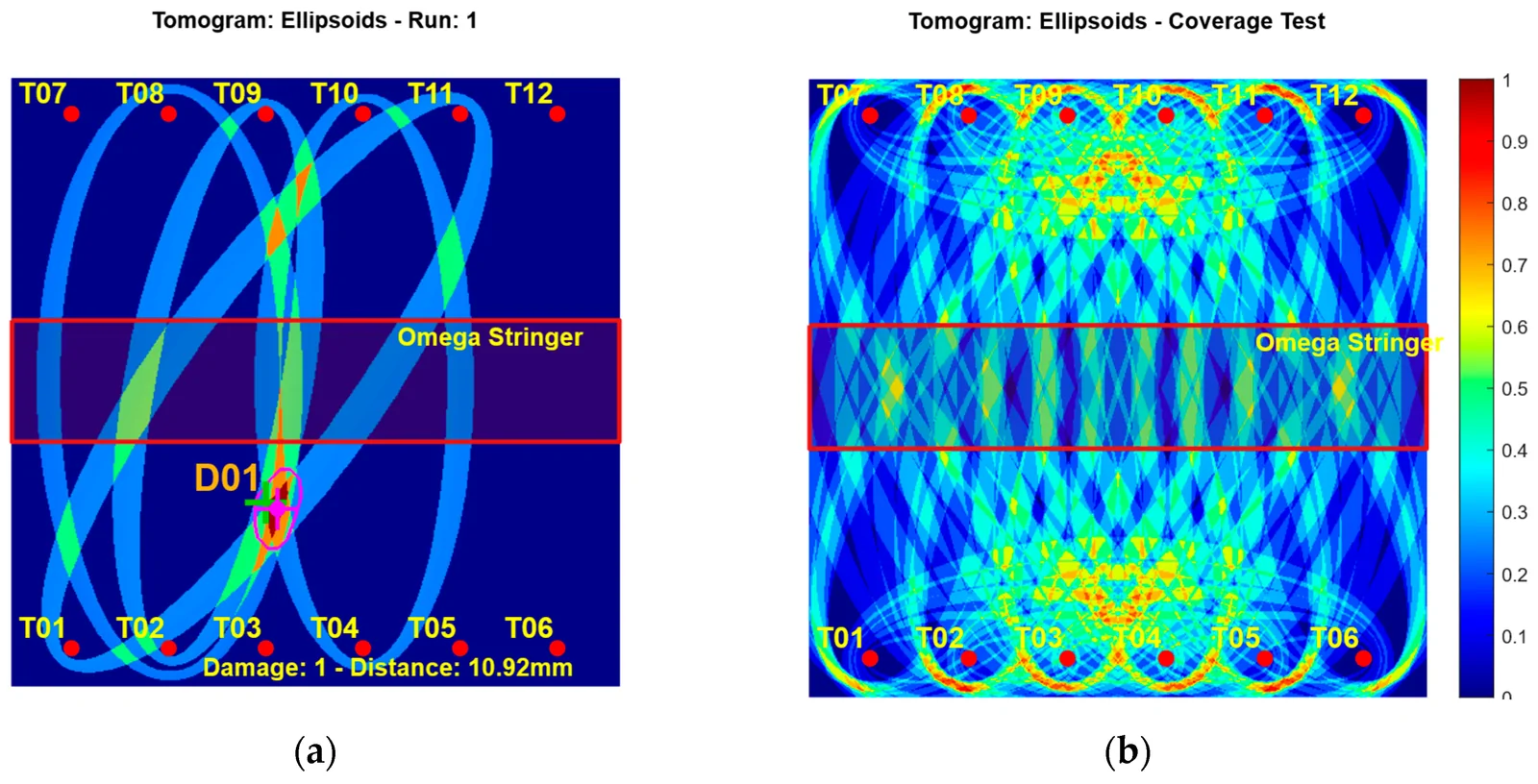
(**a**) Example localization using the ellipsoid-based method. (**b**) Corresponding coverage test showing blind spots in the localization tomogram.

The second localization method is based on plotting ellipsoids reconstructed from the predicted quantized diameter ratios following Equation (7). The damage location is estimated as the intersection region of the corresponding ellipsoids [24]. Figure 14 shows an example of damage localization using this approach. Although this method yields a lower localization error, the coverage test reveals blind spots (areas shown in dark blue), which may reduce localization reliability.

(7) $$E_{i,j}\left(x,y\right)=\left\{\begin{array}{cc} R_{\emptyset } & for\;\left|{\beta -R}_{\emptyset }\right|<∆\\ 0 & \;else \end{array}\right.$$

E: Reconstructed tomogram based on ellipsoids
$R_{\emptyset }$: Diameter ratio
i,j: Indices of the transmitter-receiver pair
x,y: Cartesian coordinates on the SHM plate
β: Thresholding parameter indicating the damage location
∆: Small value of 0.01 to include damages that lie on the perimeter of the ellipsoid

The third method reconstructs the tomogram by employing the normal distribution approach [43]. The main purpose of this approach is to combine the advantages of the previous methods by providing continuous and smoother spatial coverage of the SHM structure while maintaining low localization error. To achieve this, the damage probability inside each sensor-pair ellipse is estimated using a normal distribution, as defined by Equation (8). The employed standard deviation controls the spatial spread of the probabilistic reconstruction. Smaller standard deviation values produce narrower probability distributions and therefore sharper localization, whereas larger standard deviation values increase the spatial coverage by smoothing the reconstructed tomogram at the expense of localization precision.

**Figure 15:**
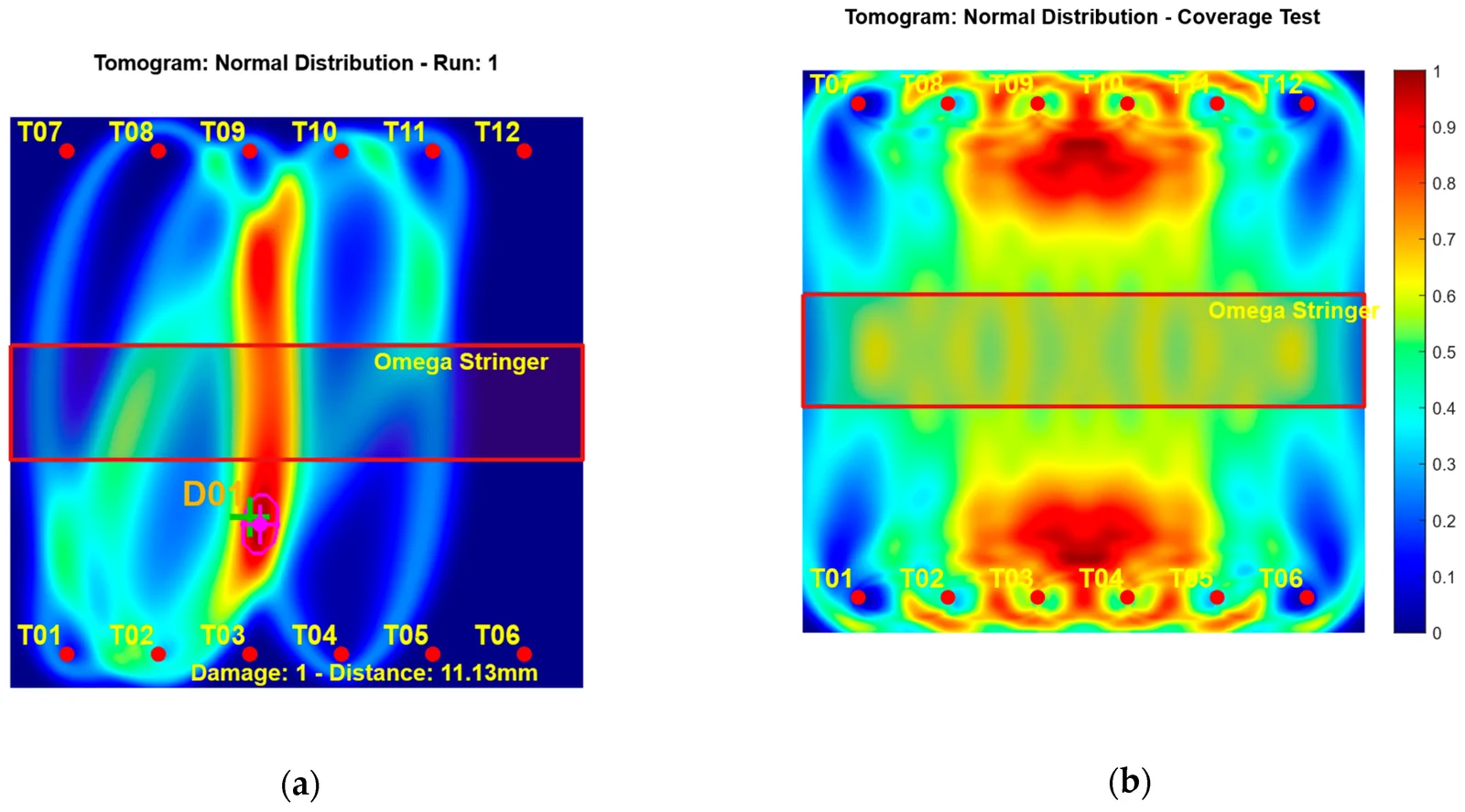
(**a**) Example localization using the normal distribution. (**b**) Corresponding coverage test of the method demonstrating the coverage of the full area.

Figure 15 illustrates the localization result for the same example considered in the previous methods. The corresponding coverage test demonstrates full-area coverage achieved with this approach.

(8) $$N_{i,j}\left(x,y\right)=\frac{e^{-{\left(R_{\emptyset }-\mu \right)}^{2}/{2\sigma }^{2}}}{\sqrt{2\pi }\sigma }$$

N: Reconstructed tomogram based on normal distribution
$R_{\emptyset }$: Diameter ratio
i,j: Indices of the transmitter-receiver pair
x,y: Cartesian coordinates on the SHM plate
μ: Quantized diameter ratio $R_{\emptyset }$ predicted by the SVM for the respective sensor pair
σ: Standard deviation set to $10^{-3}$ to achieve the intended spatial spread and area coverage

## 4. Results

In this section, the evaluation of the considered dataset is presented. First, an appropriate segmentation length is determined. Subsequently, the localization results for each method are shown, followed by a comparison of the different localization approaches.

### 4.1. Description

To identify the optimal segmentation length, values ranging from 1 (representing sample-wise comparison between baseline and damaged signals) up to 2000 were evaluated. Rather than a linear sweep, 125 discrete configurations were selected by prioritizing unique segmentation counts. Specifically, to optimize evaluation speed, segmentation lengths that yielded an identical number of segments were excluded, as they provide redundant information.

**Figure 16:**
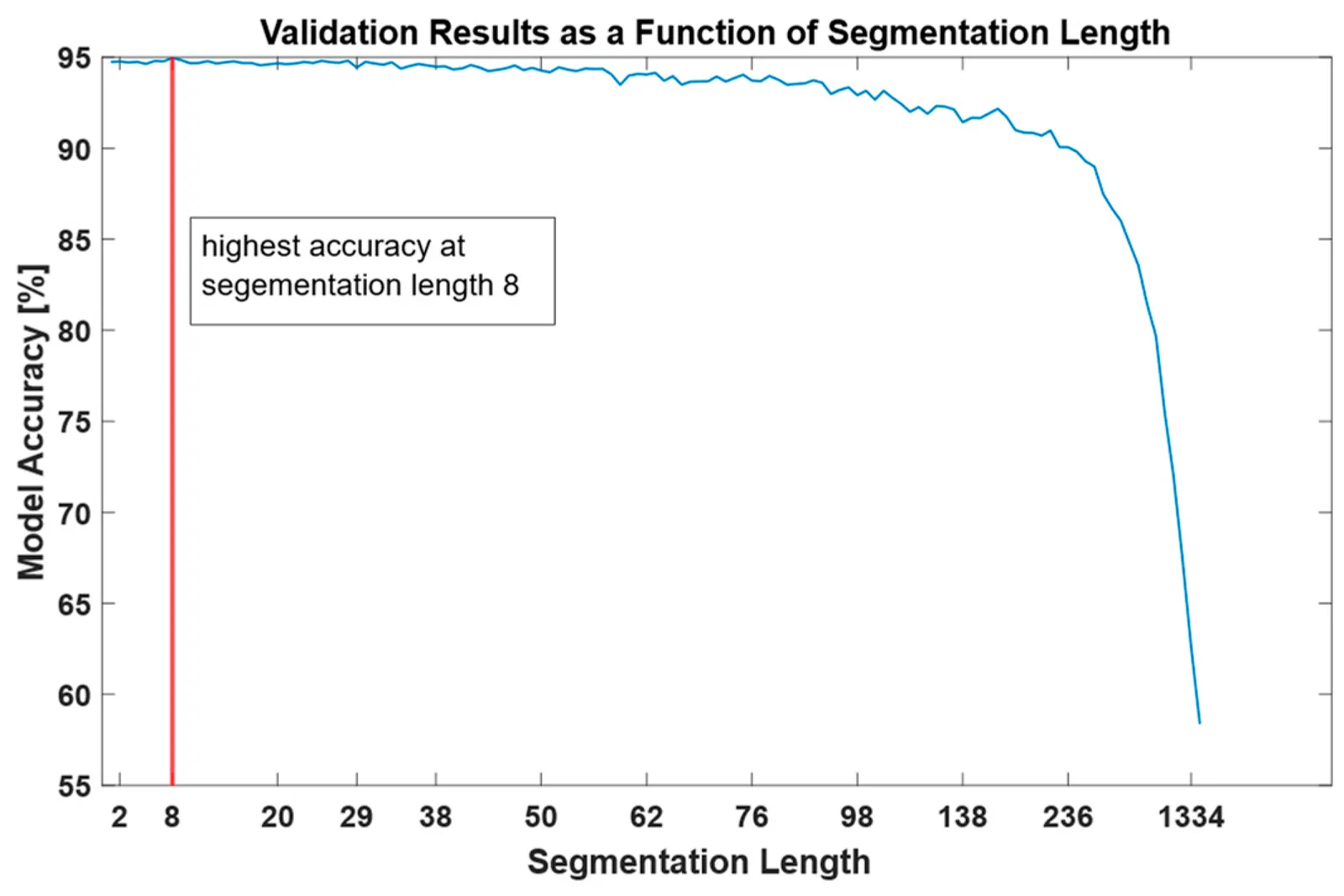
Model accuracy as a function of segmentation length. The highest accuracies are achieved for segment lengths between 1 and 28.

The evaluation results following the processing pipeline in Figure 12, indicate higher model accuracy for lower segmentation lengths in the range of 1 to 28 (Figure 16). Beyond this range, the accuracy decreases as the segmentation length increases. The accuracy is calculated by comparing the predicted quantized diameter ratios with the corresponding target diameter ratios [24]. Within the stable interval from 1 to 28, the highest accuracy of 94.95% is achieved for a segmentation length of 8. Therefore, this segmentation length is selected for the subsequent evaluation and comparison of the localization methods considered in this study.

### 4.2. Elliptical Distribution Localization

**Figure 17:**
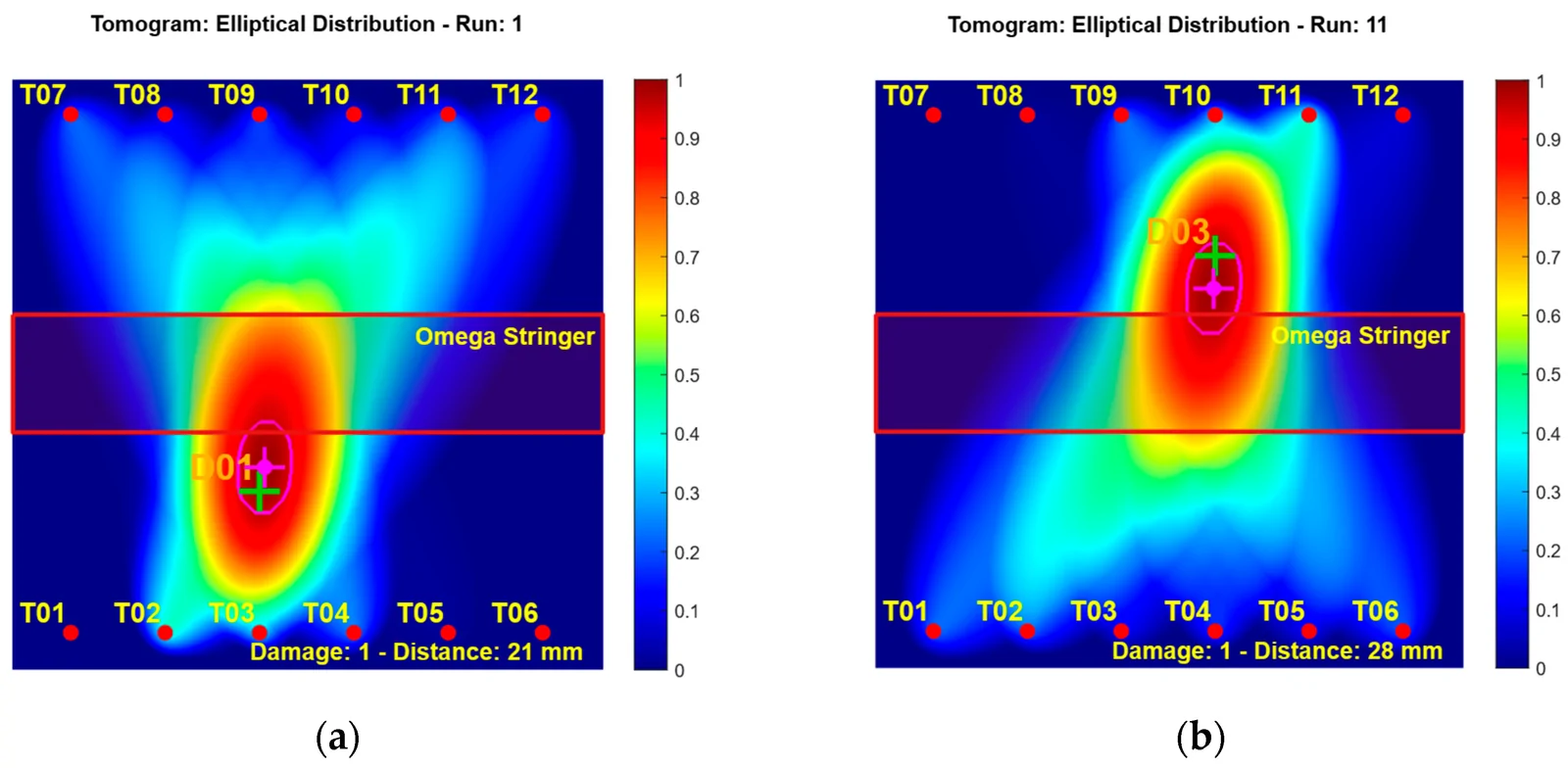
(**a**) Tomograms reconstructed using the elliptical-distribution-based localization for different damage locations at damage size 13. (**b**) The green and magenta plus symbols indicate the true and predicted damage locations, respectively.

Figure 17 shows the prediction results for the elliptical distribution method. The centroid of the region with the highest intensity is defined as the predicted damage location and is marked with a magenta plus symbol, while the true damage location is indicated by a green plus symbol for comparison. The localization error is reported below each tomogram. Table 1 provides a complete overview of the localization error obtained using this method.

### 4.3. Ellipsoid Localization

**Figure 18:**
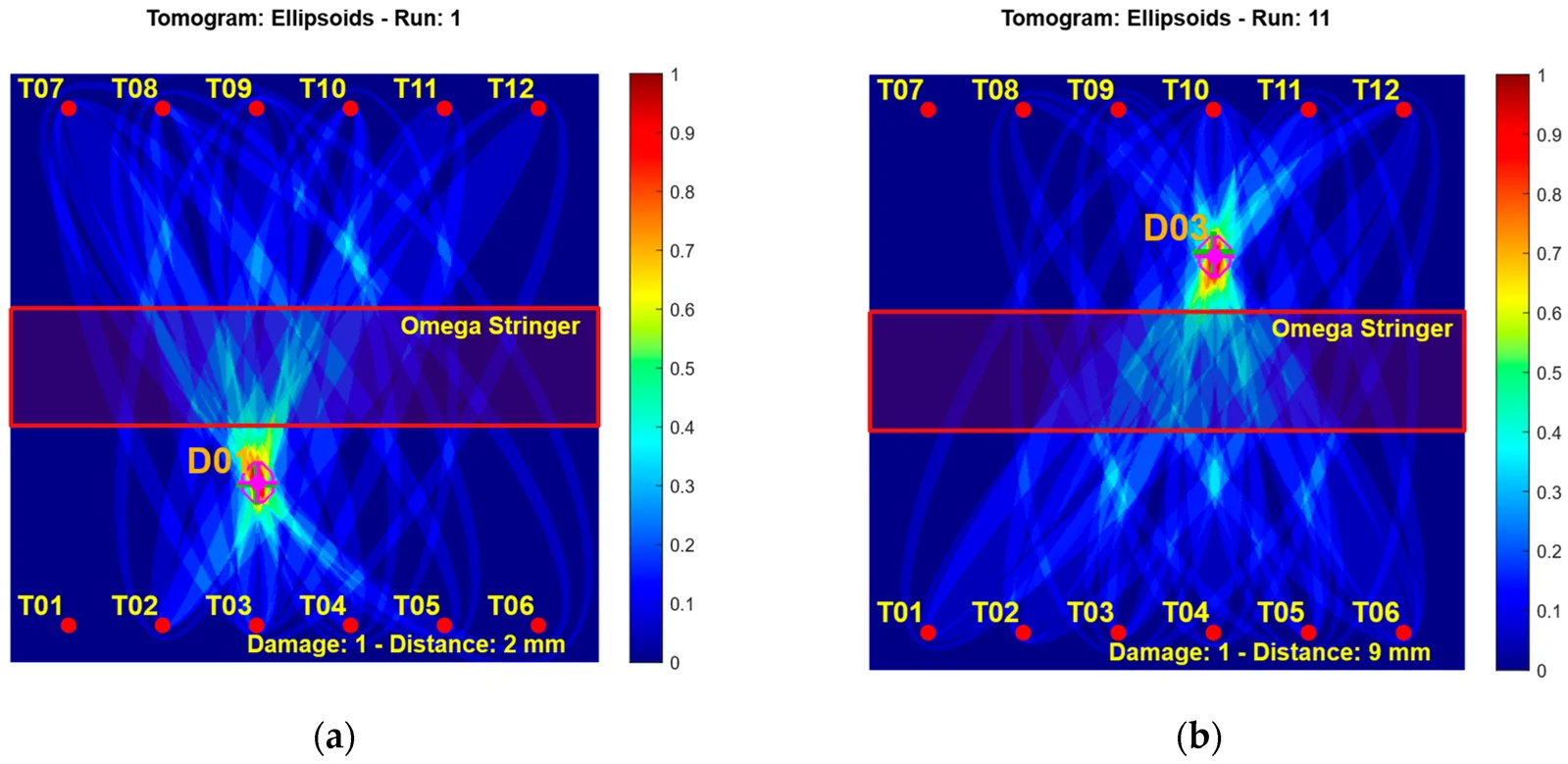
(**a**) Tomograms reconstructed using the ellipsoid-based localization for different damage locations at damage size 13. (**b**) The green and magenta plus symbols indicate the true and predicted damage locations, respectively.

For the ellipsoid-based localization, the predicted damage location is again marked with a magenta plus symbol, and the true damage location with a green plus symbol (Figure 18). The detailed localization results obtained with this method are summarized in Table 2.

### 4.4. Normal Distribution Localization

**Figure 19:**
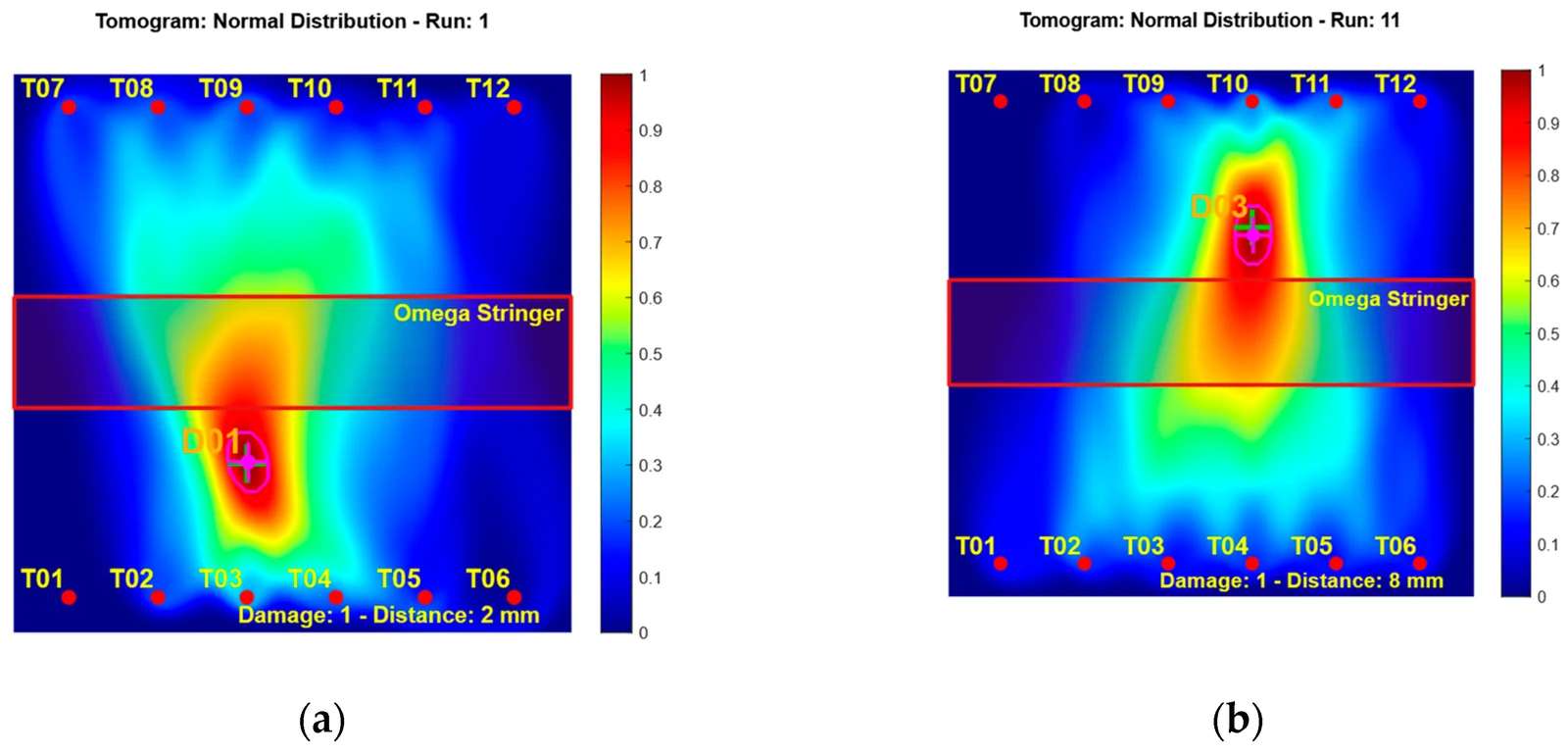
(**a**) Tomograms reconstructed using the normal-distribution-based localization for different damage locations at damage size 13. (**b**) The green and magenta plus symbols indicate the true and predicted damage locations, respectively.

Figure 19 illustrates the tomograms generated using the normal distribution-based localization. The corresponding localization results are summarized in Table 3.

### 4.5. Sensitivity Analysis of the Standard Deviation Parameter

The standard deviation of the normal distribution controls the spatial spread of the probabilistic reconstruction and therefore affects the localization performance. To evaluate its influence, the standard deviation was varied over two orders of magnitude using logarithmically spaced values from $10^{-4}$ to $10^{-2}$. The localization error was averaged over all investigated damage locations, damage sizes, and repetitions. The corresponding results are summarized in Table 4. A standard deviation of $10^{-4}$ produced the lowest localization error for successful reconstructions. However, only 79 of the 195 investigated cases yielded a valid localization result. The remaining reconstructions failed because the probability distributions became too narrow to form a common localization region. All larger standard deviation values produced valid reconstructions for every investigated case. Increasing the standard deviation beyond $10^{-3}$ gradually reduced the localization accuracy because the reconstructed tomograms became increasingly smooth. The localization performance remained relatively stable for standard deviation values between $10^{-3.5}$ and $10^{-3}$. For the investigated configuration, a standard deviation of $10^{-3}$ provided the best balance between reliable reconstruction and localization accuracy.

### 4.6. Computational Performance

To estimate the computational effort of the proposed framework, the runtime of a representative inspection cycle was measured using MATLAB R2022b (The MathWorks Inc., Natick, MA, USA) on a standard computer equipped with an Intel^®^ Core™ i5-1240P processor (1.70 GHz) and 32 GB of RAM. Table 5 summarizes the execution time of the individual processing steps. The complete inference pipeline, including NRMSD feature extraction, SVM inference, and tomogram reconstruction, required approximately 234 ms per inspection cycle. The tomogram was reconstructed on a 500 × 500 grid, corresponding to a spatial spacing of approximately 1 mm for the investigated structure. Since the proposed framework relies solely on lightweight feature extraction, SVM inference, and probabilistic reconstruction, it is well-suited for near-real-time SHM applications [44]. The reported reconstruction time mainly results from the current implementation, in which the normal-distribution map is recalculated over the full grid for each sensor path. Since the sensor geometry and possible quantized model outputs are known in advance, these spatial distributions could be precomputed and stored in a lookup table. This would reduce the online reconstruction effort and further improve the suitability of the framework for near-real-time applications. The reported runtime is hardware dependent and should therefore be regarded as a representative runtime benchmark rather than an implementation-independent performance measure.

### 4.7. Results Comparison

One of the objectives of this study is to investigate the influence of damage size on model accuracy and localization error. The localization results reported in Table 1, Table 2 and Table 3 further allow a comparison across the three investigated damage locations. Although minor variations in localization error are observed between D1, D2, and D3, no systematic dependence on the damage location can be identified for any of the investigated localization methods. Instead, all three locations exhibit the same general trend of decreasing localization error with increasing damage size, indicating that the damage size has a stronger influence on localization performance than the investigated damage position. The average localization error was calculated by aggregating the localization errors across all damage locations for a given damage size. A comparative overview of the investigated localization approaches, including their required input, baseline requirement, spatial coverage, localization accuracy, and main limitations, is provided in Table 6. The RAPID entry refers to the conventional probabilistic reconstruction concept, in particular the spatial visualization based on elliptical probability distributions. No numerical localization accuracy is reported for RAPID because, in the present study, the damage indices used for localization were obtained from the proposed ML model rather than from the conventional RAPID signal-correlation formulation.

**Figure 20:**
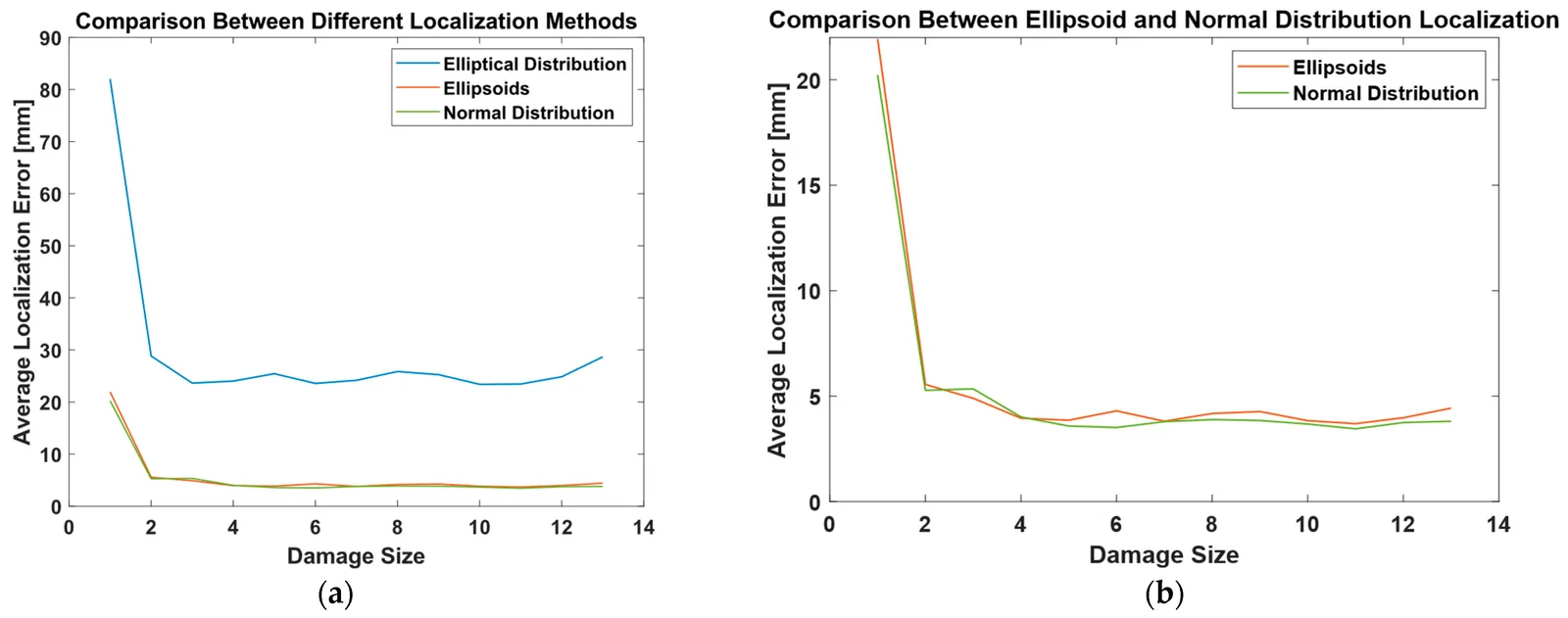
(**a**) Average localization error as a function of damage size for the investigated localization methods. (**b**) Detailed comparison of the ellipsoid- and normal-distribution-based localization methods.

Figure 20 a shows a decreasing trend in the average localization error for smaller damage sizes up to size index 4. For sizes ranging from 4 to 13, the ellipsoid and normal distribution methods exhibit low and stable localization errors. The elliptical distribution produced larger localization errors throughout the investigated damage sizes. This indicates that its spatial resolution is insufficient for accurate localization in the investigated geometry, despite the small variations observed between individual damage sizes. With the exception of damage size 3, the normal-distribution-based localization produced slightly lower localization errors than the ellipsoid-based approach (Figure 20b). The continuous probabilistic reconstruction reduces the blind regions that occur between discrete ellipsoid intersections while preserving the geometric localization information provided by the predicted diameter ratios. Damage size 3 showed a slightly larger localization error than the ellipsoid-based reconstruction. Since this behavior was not observed for the remaining damage sizes, it is most likely related to the specific distribution of the activated sensor paths or to local geometric effects rather than to a general limitation of the proposed method. Therefore, the main benefit of the proposed approach is the improved spatial coverage while maintaining localization accuracy comparable to that of the ellipsoid-based method.

## 5. Discussion

The excitation frequency of 40 kHz was intentionally selected to facilitate future comparisons with finite-element-based localization approaches operating at the same frequency. In addition, one objective of this study was to evaluate the proposed NRMSD-based localization framework independently of excitation-frequency optimization. Consequently, the excitation frequency was deliberately fixed instead of selecting the frequency that yielded the highest localization accuracy. The observed reduction in localization performance for the smallest investigated damage sizes is therefore mainly associated with the selected excitation frequency and the reduced sensitivity of individual sensor paths close to the detectability limit. At 40 kHz, the associated wavelength limits sensitivity to very small defects, consistent with previous investigations reported in [45]. Those studies demonstrated that damage sizes close to the theoretical detectability limit can still be identified; however, not every sensor path remains equally sensitive, and the resulting signal deviations may become comparable to measurement variability. For this reason, all quantitative evaluations were performed using the same excitation frequency to ensure a fair comparison across all investigated damage sizes. The results obtained in this study are therefore limited to the investigated excitation frequency of 40 kHz. The localization performance at other excitation frequencies was not evaluated. A dedicated investigation of other excitation frequencies and their interaction with damage size and geometric complexity remains an important topic for future work. The large errors observed in individual small-damage cases also result in comparatively high standard deviations. Therefore, the mean localization error should be interpreted together with the individual errors and their variability, particularly close to the detectability limit.

The experimental evaluation was carried out under laboratory conditions only. Environmental influences such as temperature variations, humidity, and operational loading were not included in this study. These factors are known to affect guided-wave propagation and may require dedicated compensation before the proposed framework can be applied in practical SHM systems. Changes in wave velocity, amplitude, or phase can increase the difference between the current signal and the baseline even without damage. This may change the NRMSD features and, consequently, the predicted path-wise damage information used for localization. The reconstructed tomogram may therefore also be affected by environmental baseline drift. Their influence was outside the scope of the present work and should be investigated separately.

The feature extraction and SVM classification stages were adopted from our previous work [24]. The methodological extension investigated here concerns the spatial reconstruction stage, where the normal-distribution-based formulation is introduced to provide continuous coverage. The second contribution is the transfer and evaluation of the complete framework on the more complex stringer-reinforced CFRP structure and across different damage sizes. Besides evaluating the localization accuracy, the experiments also showed how the individual processing stages contribute to the final result. Changing the segmentation length affected the localization accuracy, while comparing the elliptical distribution, the previously proposed ellipsoid-based reconstruction, and the proposed normal-distribution-based reconstruction made it possible to assess the contribution of each localization strategy. The results show that the final localization accuracy depends on the combination of these processing steps rather than on a single algorithm. Additional preprocessing methods and different hyperparameter settings could help to better understand the influence of each processing stage. A coarser reconstruction grid could further reduce computation time, although its influence on localization accuracy was not investigated in this study.

The investigated damage locations were selected to allow a quantitative comparison between the localization methods. In practical SHM applications, however, defects can occur at arbitrary positions. Depending on the damage location, some sensor paths intersect the damaged region directly, while others pass nearby or are only weakly affected. The final localization is therefore based on the information provided by all sensor paths instead of a single measurement. This is one of the main advantages of probabilistic localization methods.

The present LOGO-CV strategy evaluates generalization to unseen damage sizes but does not independently test unseen damage positions. Although damage-location generalization was investigated in our previous work [24], further validation for unseen positions remains necessary. Future work will address this using additional experimental data and simulation-based training.

Multiple closely spaced defects or disbonds were not investigated in this study. These damage configurations may affect guided-wave propagation differently from isolated defects and should therefore be evaluated separately. The proposed normal-distribution-based method generates a continuous tomogram rather than a single damage coordinate. For comparison with the ellipsoid-based approach, the damage position was determined from the centroid of the connected high-intensity region around the tomogram maximum. Only this centroid was used for the quantitative evaluation. The area and shape of this region were not analyzed further because this would require an additional study of the intensity threshold. The tomogram intensity is also not a statistically calibrated confidence probability. A false-alarm probability would require a separate damage/no-damage criterion and validation with undamaged measurements. These aspects should be investigated in future work.

The sensitivity analysis for Equation (8) showed that the proposed reconstruction is relatively insensitive to moderate changes of the standard deviation parameter. Although a standard deviation of $10^{-4}$ produced the lowest localization error for successful reconstructions, it also resulted in a considerable number of failed localizations because the reconstructed probability distributions became too narrow to form a common intersection region. Increasing the standard deviation beyond $10^{-3}$ gradually reduced the localization accuracy because the reconstructed tomograms became increasingly smooth. For the investigated configuration, a standard deviation of $10^{-3}$ provided the best balance between localization accuracy and reconstruction reliability. The value of σ used in this study was selected for the employed experimental setup. It may change for another sensor arrangement or another structure. In that case, several values should be tested and compared in terms of localization error and reconstruction reliability. The effect of sensor layout and structural geometry on the choice of σ should be investigated in future work. The influence of the radius cut-off parameter β was investigated in [24]. However, its interaction with the standard deviation and the segmentation length has not yet been studied systematically. This combination of parameters could be investigated further to improve the localization performance for different structural configurations.

Finally, the localization results in this study are based on isolated reversible artificial damage. Cases with multiple damages and real defects, such as delamination, cracks, impact damage, or disbonding, were not considered. These cases should be investigated in future work.

## 6. Conclusions

This study investigated the transferability of the previously proposed interpretable NRMSD-based machine-learning framework for ultrasonic guided-waves damage detection. The framework, originally developed for composite overwrapped pressure vessels, was successfully transferred to a geometrically complex CFRP structure with an integrated omega stringer, representative of aerospace composite components. The developed model achieved an accuracy of nearly 95% and an average localization error of 5 mm, demonstrating the transferability of the proposed framework.

The influence of the segmentation length was systematically investigated, showing that short segmentation lengths preserve local signal characteristics and provide the highest localization accuracy. Furthermore, the results reveal a clear relationship between damage size and localization performance, with reduced accuracy for the smallest investigated damages due to the physical detectability limit associated with the selected excitation frequency of 40 kHz.

Finally, a normal-distribution-based probabilistic localization approach was proposed. Compared with the previously developed ellipsoid-based reconstruction, the proposed method preserves continuous spatial coverage while maintaining high localization accuracy, thereby combining the principal advantages of ellipsoid-based and probabilistic tomographic localization methods.

The proposed normal-distribution-based reconstruction extends the applicability of the interpretable NRMSD framework by combining high localization accuracy with continuous spatial coverage, thereby providing a promising framework for structural health monitoring of complex composite aerospace structures.

## Figures and Tables

**Table 1:** Localization Error Using The Elliptical Distribution Approach For All Damage Sizes And Locations. Average Error Across All Sizes Is 29.5 mm.

Damage Location D_1–3_ and Repetition 1–5		Average Error ± Standard Deviation for Each Damage Location in mm	Localization Error for Each Damage Size 1–13 in mm												
			1	2	3	4	5	6	7	8	9	10	11	12	13
D1	1	30.4 ± 19.9	18	25	26	26	27	24	26	29	26	26	26	24	21
	2		58	20	26	31	29	26	26	31	24	26	24	26	18
	3		148	40	23	29	30	26	30	26	26	24	26	26	23
	4		99	19	29	24	26	26	26	26	23	24	26	26	18
	5		93	26	26	26	29	30	26	33	26	29	26	26	23
D2	1	27.5 ± 31.6	156	41	17	17	16	16	17	17	10	17	19	17	35
	2		24	18	19	17	38	16	17	16	53	17	17	17	38
	3		171	17	17	17	16	17	17	17	17	17	17	38	18
	4		156	37	17	17	16	17	18	16	17	16	17	17	14
	5		24	17	17	17	16	17	17	38	17	17	17	17	83
D3	1	30.6 ± 8.6	76	28	25	28	28	28	28	28	28	28	28	28	28
	2		37	40	28	28	28	28	28	28	28	28	28	28	30
	3		51	40	28	29	28	28	28	28	28	28	26	28	30
	4		61	38	28	28	28	28	31	28	28	28	28	28	28
	5		56	28	28	28	28	28	28	28	28	28	28	26	24
**Average Error in mm:**		**29.5 ± 22.1**	81.9	28.9	23.6	24.1	25.5	23.7	24.2	25.9	25.3	25.3	25.3	24.8	28.7

**Table 2:** Localization Error Using The Ellipsoid-Based Approach For All Damage Sizes And Locations. Average Error Across All Sizes Is 5.5 mm.

Damage Location D_1–3_ and Repetition 1–5		Average Error ± Standard Deviation For Each Damage Location in mm	Localization Error for Each Damage Size 1–13 in mm												
			1	2	3	4	5	6	7	8	9	10	11	12	13
D1	1	7.0 ± 18.5	12	9	7	4	3	9	3	2	4	3	3	6	2
	2		19	7	7	3	3	3	3	3	5	3	3	3	3
	3		150	15	4	2	2	3	3	2	3	3	3	3	7
	4		6	7	7	7	3	3	3	4	3	3	3	3	3
	5		26	4	3	3	3	4	3	3	4	3	3	3	3
D2	1	4.1 ± 4.5	9	3	3	3	3	3	3	3	6	3	3	3	3
	2		18	3	3	3	3	3	3	3	3	3	3	3	3
	3		35	3	3	3	3	3	3	3	3	3	3	3	5
	4		6	3	3	3	3	3	3	10	3	3	3	3	4
	5		6	3	3	3	3	3	3	3	3	3	3	3	3
D3	1	5.5 ± 1.9	4	5	5	5	5	5	5	5	5	5	5	5	9
	2		17	5	5	5	5	5	5	5	6	6	6	5	5
	3		7	5	9	4	5	5	5	5	5	5	3	5	4
	4		4	6	5	5	5	5	6	5	5	5	5	5	3
	5		11	5	5	5	7	5	5	5	5	5	5	6	8
**Average Error in mm:**		**5.5 ± 11.0**	22.0	5.5	4.8	3.9	3.7	4.1	3.7	4.1	4.2	3.7	3.6	3.9	4.3

**Table 3:** Localization error using normal distribution-based approach for all damage sizes and locations. Average error across all sizes is 5.3 mm.

Damage Location D_1–3_ and Repetition 1–5		Average Error ± Standard Deviation for Each Damage Location in mm	Localization Error for Each Damage Size 1–13 in mm												
			1	2	3	4	5	6	7	8	9	10	11	12	13
D1	1	6.2 ± 15.7	20	6	6	4	3	2	3	4	3	3	3	5	2
	2		10	1	6	4	2	3	3	2	3	3	2	3	2
	3		128	14	4	3	2	3	2	3	3	3	3	3	6
	4		4	13	6	7	4	3	3	5	3	3	3	3	5
	5		18	3	3	3	4	3	4	2	3	3	2	3	3
D2	1	4.3 ± 8.5	3	3	3	3	3	3	3	3	5	3	1	3	2
	2		14	3	4	3	2	2	3	4	3	3	3	3	3
	3		71	3	4	3	3	3	3	3	3	3	3	3	3
	4		3	3	3	3	2	3	3	5	3	3	3	3	3
	5		4	3	3	3	3	3	3	3	3	3	3	3	4
D3	1	5.3 ± 1.7	3	5	6	5	5	5	5	5	5	5	5	5	8
	2		10	5	5	5	5	5	5	5	5	6	5	5	4
	3		5	5	16	5	5	5	5	5	5	5	5	5	5
	4		5	7	5	5	5	5	7	5	5	5	5	5	1
	5		5	5	5	5	6	5	5	5	5	5	6	5	7
**Average Error in mm:**		**5.3 ± 10.0**	20.2	5.3	5.3	4.1	3.6	3.5	3.8	3.9	3.8	3.7	3.5	3.8	3.9

**Table 4:** Robustness analysis of the standard deviation parameter.

Standard Deviation σ	Valid Reconstructions	Failure Rate	Mean Error ± Standard Deviation [mm]
10^−4^	79/195	59%	4.4 ± 5.3
10^−3.5^	195/195	0%	4.9 ± 8.1
10^−3^	195/195	0%	5.3 ± 10.4
10^−2.5^	195/195	0%	7.8 ± 10.6
10^−2^	195/195	0%	14.6 ± 14.3

**Table 5:** Computational Performance Of The Proposed Framework.

Processing Step	Runtime [ms]
NRMSD feature extraction	8.88
SVM inference	5.30
Normal-distribution tomogram reconstruction	220.00
Total	234.18

**Table 6:** Comparison of the investigated localization approaches in terms of required input, baseline requirement, spatial coverage, localization accuracy, and main limitations.

Method	Required Input	Baseline Required	Spatial Coverage	Localization Accuracy	Main Limitation
RAPID/elliptical probability reconstruction	Path-wise damage indicator, typically signal correlation	Yes	Continuous with diameter cutoff	Comparatively diffuse localization	Spatial resolution depends strongly on sensor geometry and spatial weighting
Ellipsoid-based localization [24]	ML-predicted diameter ratio for each sensor path	Yes	Contains blind areas	High; 5.5 mm average error in this study	Blind areas occur between ellipsoid intersections
Proposed normal-distribution localization	ML-predicted diameter ratio for each sensor path	Yes	Continuous/full-area coverage	High; 5.3 mm average error in this study	Spatial spread depends on the selected standard deviation

## Data Availability

The contributions of this study are provided within the article. For additional information or questions, please contact the correspondence author.

## Abbreviations

The following abbreviations are used in this manuscript:

SHM	Structural Health Monitoring
NDT	Nondestructive Testing
UGWs	Ultrasonic Guided Waves
RAPID	Reconstruction Algorithm For Probabilistic Inspection Of Damage
ML	Machine Learning
NRMSD	Normalized Root Mean Square Deviation
CFRP	Carbon Fiber-Reinforced Polymer
RMSE	Root Mean Square Error
SVM	Support Vector Machine
LOGO-CV	Leave-One-Group-Out Cross-Validation
